# Designing Persuasive Food Conversational Recommender Systems With Nudging and Socially-Aware Conversational Strategies

**DOI:** 10.3389/frobt.2021.733835

**Published:** 2022-01-19

**Authors:** Florian Pecune, Lucile Callebert, Stacy Marsella

**Affiliations:** ^1^ Institute of Neuroscience and Psychology, University of Glasgow, Glasgow, United Kingdom; ^2^ Khoury College of Computer Sciences, Northeastern University, Boston, MA, United States

**Keywords:** health aware, food recommender systems, conversational agents, nudging, socially aware

## Abstract

Unhealthy eating behavior is a major public health issue with serious repercussions on an individual’s health. One potential solution to overcome this problem, and help people change their eating behavior, is to develop conversational systems able to recommend healthy recipes. One challenge for such systems is to deliver personalized recommendations matching users’ needs and preferences. Beyond the intrinsic quality of the recommendation itself, various factors might also influence users’ perception of a recommendation. In this paper, we present Cora, a conversational system that recommends recipes aligned with its users’ eating habits and current preferences. Users can interact with Cora in two different ways. They can select pre-defined answers by clicking on buttons to talk to Cora or write text in natural language. Additionally, Cora can engage users through a social dialogue, or go straight to the point. Cora is also able to propose different alternatives and to justify its recipes recommendation by explaining the trade-off between them. We conduct two experiments. In the first one, we evaluate the impact of Cora’s conversational skills and users’ interaction mode on users’ perception and intention to cook the recommended recipes. Our results show that a conversational recommendation system that engages its users through a rapport-building dialogue improves users’ perception of the interaction as well as their perception of the system. In the second evaluation, we evaluate the influence of Cora’s explanations and recommendation comparisons on users’ perception. Our results show that explanations positively influence users’ perception of a recommender system. However, comparing healthy recipes with a decoy is a double-edged sword. Although such comparison is perceived as significantly more useful compared to one single healthy recommendation, explaining the difference between the decoy and the healthy recipe would actually make people less likely to use the system.

## 1 Introduction

Unhealthy eating is a major public health burden worldwide that has serious personal risks in terms of health outcomes and costs. At the core, this behavior can be managed through behavior change counselling in labour-intensive individual or group sessions with therapists or dietitians (Webb and Sheeran, 2006). Such approaches, however, are very costly in terms of time and money, and are not scalable to the current epidemic of obesity. At the public level, the common approach to persuade people to change their food consumption behavior is to inform them about what a healthy diet should be. Nevertheless, informing people is not always sufficient to trigger behavior change: food taste, habits, price, or convenience are all important factors people take into account when they decide what to eat (Guthrie et al., 2015).

Rather than convincing people to change their habits, researchers introduced the notion of *nudging*, a method to indirectly alter people’s decision towards more desirable options without interfering with their freedom of choice (Thaler et al., 2008). Nudges can take many different forms (Caraban et al., 2019) and have been proven to be effective in various domains (Hummel and Maedche, 2019). Given the complex nature of food related decisions (Cunningham and Bainbridge, 2013), nudging applies particularly well to healthy eating (Tørris and Mobekk, 2019).

A common strategy to nudge people towards healthy food consumption is to play with products’ positioning (Bucher et al., 2016). For instance, people are more likely to buy a healthy candy bar when placed in between two less healthy options in a shelf (Keller et al., 2015). A field study at a train station demonstrated that shops sold more healthy products when positioning them at the cash register desk while keeping unhealthy options available elsewhere (Kroese et al., 2016). Besides positioning, food choices can also be manipulated by making healthy options more convenient to select on a menu (Downs et al., 2009), by dedicating a lunch line to healthy food (Hanks et al., 2012), or by explicitly stating the scarcity on specific products (Fennis et al., 2020).

In parallel with these real-life applications, online behavior also needs to be investigated. Indeed, people often search for inspiration online when choosing what they want to eat (Yasukawa et al., 2014), However, most of the popular recipes found on the web are unhealthy (Trattner and Elsweiler, 2017b). One solution to overcome this issue and help people make healthier choices is to develop health-aware food recommender systems (Trang Tran et al., 2018). Such systems alleviate the choice overload due to the abundance of options (Scheibehenne et al., 2010) by recommending healthy recipes matching people preferences. One trend to improve users’ experience when interacting with a recommender system is to design the recommendation process as a conversation. A theoretical framework of conversational search is exposed in (Radlinski and Craswell, 2017) which determines when, why, and how to use a conversational approach to query information. Besides helping users to achieve task-oriented goals, conversations can also fulfill interpersonal functions, such as building rapport (Tracy and Coupland, 1990). Rapport can be described as a dynamic process that can be achieved when people “click” with each other or feel the interaction is due to “chemistry” (Tickle-Degnen and Rosenthal, 1990). Human-human studies have found that rapport between two people can influence task performance in situations as diverse as peer-tutoring (Sinha and Cassell, 2015) and negotiation (Drolet and Morris, 2000). These findings suggest that endowing recommender systems with social conversational abilities would build rapport with users and improve task effectiveness.

One of the most effective strategy to build rapport during a conversation is to reveal personal information about oneself (Zhao et al., 2014). This communication process of revealing personal information to somebody else is called *self-disclosing* (Greene et al., 2006). Self-disclosing information during an interaction has been linked to affiliative interpersonal outcomes such as liking (Collins and Miller, 1994) and trust (Moon, 2000), and is also known to have a positive impact in domains such as negotiation or sales outcome (Jacobs et al., 2001). Hence, for conversational agents to build rapport using self-disclosures, they need to have a believable and coherent backstory to disclose (Simmons et al., 2011). However, the use of backstories during human-computer interactions raises some issues. Indeed, systems pretending to be humans might become distrusted once users discover they are not interacting with a real human (Bickmore, 2005).

In this paper, we present a conversational system able to recommend recipes matching users’ needs while building rapport with them. More specifically, our work focuses on investigating how the conversational skills of a recipe recommender system and the nudges it relies on would influence users’ perception and their intention to cook. First, we describe the design of our system and its architecture before we explain how the recommendation process works. Then, we evaluate our system through two experiments with real users. In the first experiment, we study the impact of our system’s conversational skills and interaction mode on its persuasiveness and on users’ experience. In the second experiment, we evaluate whether recipe comparisons and recommendation explanations both have a significant impact on users’ perception of a healthy recipe recommender system.

## 2 Related Work

### 2.1 Food Recommender Systems

Food recommender systems mostly rely on three distinct approaches to recommend recipes matching their users’ needs (Trang Tran et al., 2018). In the *content-based* approach (CB), systems first build a user profile by inferring relevant features from recipes liked by the user. These features can be ingredients and cuisine types (Freyne and Berkovsky, 2010a), dietary information (Yang et al., 2017), or fine-grained tags (Khan et al., 2019). The system then relies on this user profile to deliver subsequent recommendations. Instead of inferring features from recipes, systems relying on the *knowledge-based* approach (KB) directly ask users which features they like. For example, in the goal-oriented recommender system from (Ueta et al., 2011), the system first collects the user’s goal before finding a nutrient that matches that goal. The system then recommends the recipe containing the most of the nutrient previously selected. One limitation of content-based and knowledge-based approaches is that their performance highly depends on the quality of the features populating the user profile. Building such systems hence requires a lot of domain knowledge.

Systems relying on the *collaborative filtering* approach (CF) try to overcome this limitation by predicting recommendation ratings for a user based on ratings from other users. In (Ge et al., 2015a), the authors developed a system that first collects users’ preferences by asking them to rate and tag the recipes they usually cook at home. The system then relies on ratings from similar users to rank recipes and deliver recommendations. Authors found that their improved matrix factorization algorithm outperformed the content-based approach proposed by (Freyne and Berkovsky, 2010a). The extensive comparison performed in (Trattner and Elsweiler, 2019) confirms that collaborative filtering approaches perform better than content-based ones. These techniques can be combined in what is called a hybrid approach. CB and CF techniques are for example combined in (Freyne and Berkovsky, 2010b), while the evaluation performed in (Burke, 2002) shows the advantages of combining KB with CF techniques.

Most of these systems solely focus on delivering personalized recommendations without considering the healthiness of the recipes. Significant effort has been put recently into tailoring food recommendation algorithms to reconcile users’ preferences with healthy recommendation. One solution is to bias the predicted ratings from the collaborative filtering algorithm with a *“health weight”*. This weight can for example represent the difference between the calories that the user needs and the calories of the recipes (Ge et al., 2015b), or the health score associated with the recipes^1^ (Trattner and Elsweiler, 2017b). The healthier the recipe, the more likely it is to be recommended. Another system reconciling healthiness and personalization is described in (Chen et al., 2020), where the authors propose a method to recommend healthy recipes based on a subset of ingredients given by a user. The system first selects ingredients that are compatible with the given subset, and associates an optimal quantity for each of these ingredients. The system then generates a pseudo-recipe containing the ingredients with the healthiest nutritional value, before picking the existing recipe best matching the pseudo-recipe. Other examples are PREFer (Bianchini et al., 2017) and DietOS (Agapito et al., 2018) which both manage specific health conditions by relying on their own knowledge base to recommend food matching their users’ pathology.

Although these systems present interesting approaches to reconcile healthiness with users’ preferences, most of them are not evaluated by real users. Furthermore, the study presented in (Trattner and Elsweiler, 2019) hints at consistency in user habits in terms of the healthiness of recipes they choose. This emphasizes the growing need to endow health-aware food recommender systems with persuasive capabilities.

### 2.2 Persuasive Systems

Persuasive systems are intentionally designed to affect people’s attitudes and behaviors (Fogg, 1998). Over the years, several design strategies have been developed to endow systems with persuasive capabilities (Oinas-Kukkonen and Harjumaa, 2008). These strategies have different modalities, as well as psychological and behavioral outcomes (Hamari et al., 2014).

One specific line of work draws on ideas from behavioral economics, more specifically on the idea of nudges, to influence people’s online behavior (Weinmann et al., 2016). Such influence can be achieved simply by adapting user interface designs without interfering with users’ options (Mirsch et al., 2017). Different nudges have different impacts depending on their domain of application. For example, triggering people’s fear and loss aversion by explicitly highlighting the risks associated with lazy password management has been proven effective (Kankane et al., 2018) in the area of cyber security. In the domain of social media services, negatively framed social nudges (e.g., “90% of users would not do that”) were the most effective strategies (Masaki et al., 2020), highlighting people’s desire to consent with others’ behavior. The decoy effect (Bateman et al., 2008) has been particularly studied during online shopping experiments. The decoy effect is a cognitive bias in which people will change their preferences between two options when also presented with a third option inferior in all respects to one of the options. The study presented in (Fasolo et al., 2006) illustrates the decoy effect by presenting participants with three different laptops: a target T with medium quality but low price, a competitor C with high quality but high price, and a decoy D, with low quality and medium price. When presented with only two options, the target T and the competitor C, participants took their decisions based on their preferences: some preferred the target T because they valued the price over the quality; others preferred the competitor C because they considered that quality was more important. However, participants were more likely to purchase the target laptop T when it was positioned in between the competitor C and the decoy D, an option objectively inferior to the target T (i.e., lower quality and more expensive).

Closer to our work, digital nudges have also been applied to the food domain to encourage people to select healthy options online. Similar to (Fasolo et al., 2006), the system described in (Lee et al., 2011) relies on the decoy effect to increase the preference for apples over less healthy options by presenting a smaller and less appealing apple next to a big shiny one. Another example demonstrating the importance of alternative options is described in (Elsweiler et al., 2017). Rather than recommending a single healthy recipe, the recommender system pairs specific recipes with similar ones containing healthier substituted ingredients. When presented with these two alternatives, people were more likely to select the healthier recipe of the two.

Other factors might have an impact on users’ experience with recommender systems, and can therefore influence users’ perception of a recommended item (Jugovac and Jannach, 2017). The way the recommendations are presented (Nanou et al., 2010) as well as the system’s embodiment (Herse et al., 2018), latency (Tsai et al., 2019), and sentence length (Pecune et al., 2018) might all affect users’ perception.

One of the most important factors is the type of explanation used by the system to justify a recommendation (Nunes and Jannach, 2017). A system can for example justify its decision by highlighting specific features users like, or similar items they previously selected (Symeonidis et al., 2009). While personalized explanations generally improve users’ satisfaction, they have a different impact on the system’s perceived effectiveness and efficiency (Gedikli et al., 2014). The authors of (Kunkel et al., 2019) compare the performance of automatically generated explanations versus human generated explanations. Their results show that higher-quality explanations coming from real humans had a positive impact on users’ trust, despite the lower perceived quality of the recommendations. Furthermore, the study in (Pecune et al., 2019) shows that people interacting with a system able to express its “own” opinion and talk about its “own” experience were more satisfied with the recommended items and had a better overall experience. Many recommender systems deliver a list of Top-N recommended items rather than a single item (Tintarev and Masthoff, 2007). In (Pu and Chen, 2007), the system separates the top item from the rest of the list, and regroup the rest in different categories. Each category represents how items compare to the top item in terms of features trade-off (e.g., “All the items in this category are less X but more Y than the top item”). In such a context, highlighting the trade-offs between the top item and other groups of items significantly improved system’s perceived competence and users’ intention to reuse the system.

### 2.3 Rapport-Building Conversational Systems

As pointed out by (Radlinski and Craswell, 2017), one way to improve users’ experience during complex search settings is to endow recommender systems with conversational skills. One of the first attempts comes from (Carenini et al., 2003) which provides a set of task-oriented techniques that a conversational recommender system can use to query user information or elicit user feedback, and describes the effect of these techniques on the system’s accuracy and user’s effort. Another formalization is proposed in (Wärnestål et al., 2007), where the authors analyze a corpus of interactions between human recommenders and users to derive two main recommendation phases: Interview and Delivery. The interview phase consists in a sequence of questions whose purpose is to gather relevant preferences from the user. The goal of the delivery phase is to actually deliver the recommendation—or a list of recommendations—based on the information gathered during the interview phase, with optional justifications or explanations. Finally, after presenting the user with a recommendation, the system must be prepared to respond to the user’s reactions, of which there can be many types. For instance, the user may want to change their preferences, specify whether they think an item is interesting or not—in which case the system must update the user’s preferences accordingly –, or if they would like to see more similar items (Tintarev and Masthoff, 2007). The approach presented in (Christakopoulou et al., 2016) optimizes the interview gathering phase by using offline data to initialize online learning recommendation systems, which reduces the number of questions that the system needs to ask before delivering a relevant recommendation. Reinforcement Learning can also be used to optimize the system’s conversational policy and select the most appropriate task-oriented strategy based on the state of the interaction (Mahmood and Ricci, 2009). More recently, (Zhang et al., 2018), proposed a personalized multi-memory network architecture to teach a recommender system which questions to ask users to deliver the most accurate recommendation.

Rather than focusing on a system’s dialogue policy or strategies, some other work tries to find the best way for users to interact with a recommender system. The authors in (Narducci et al., 2019) investigate different interaction modes by objectively comparing three versions of a same music recommender systems. The first one used buttons to interact with users, the second one used free text, and the last one was a hybrid version that used buttons whenever there was a need to disambiguate. Results show that the hybrid version had a better interaction cost and recommendation accuracy than the two other versions. All these systems aim at fulfilling the task-oriented goals of a conversation. However, this is not the only type of goal that people want to achieve during an interaction (Tracy and Coupland, 1990), and interpersonal goals such as building and maintaining a good relationship or rapport should also be considered when designing conversational systems.

Researchers have already started to investigate rapport-building conversational systems in different contexts, and study how to combine rapport-building and task-oriented strategies during an interaction (Pecune et al., 2013; Zhao et al., 2018b; Pecune and Marsella, 2020). With Rea the virtual Real Estate Agent, researchers investigated how small-talk influenced the price users were ready to invest in a new house (Cassell and Bickmore, 2003). The robotic museum guide Tinker demonstrated that users were more likely to retain information about the museum exhibits when it used predetermined rapport-building strategies during the conversation (Bickmore et al., 2013). Ellie, the virtual human interviewer for healthcare decision support, relied on non-verbal behavior and a set of dialogue policies to build rapport with its users, which make them more comfortable sharing information. In (Zhao et al., 2018a), the authors investigated rapport building strategies in the context of a non-collaborative negotiation task. Results show that in such a context, the outcome of the task had little or no impact on the rapport built during the interaction. Closer to our current work, some researchers specifically focused on rapport-building conversational agents in the context of a recommendation task. The authors of (Lee and Choi, 2017) evaluated the impact of self-disclosures and reciprocity on a conversational recommender system’s perceived performance. The results of their experiment showed that both self-disclosures and reciprocity had a significant positive impact on users’ satisfaction with the interaction, and intention to use the system. However, their system was not fully autonomous and they did not try to measure the impact of a system’s self-disclosures on the perceived quality of the recommendation. Recently, (Pecune et al., 2019), presented a conversational recommender system able to draw from the various explanations humans use with one another. The authors demonstrated that users preferred movie recommendations coming from a system that was able to justify its choice using its “own” personal opinion and talk about its “own” personal experience related to the recommended movie. In (Pecune et al., 2018), the authors deployed a socially-aware recommender assistant in a conference that was delivering recommendations while building rapport with its users. The agent was able to adapt its rapport-building conversational strategies depending on the strategies it detected from the user.

Although all these works rely on rapport-building conversational strategies, few of them investigate how rapport influences the perceived quality of the items recommended, or people’s compliance with the recommendations. Moreover, they do not investigate whether the way users interact with the system have an impact on rapport and/or on users’ perception of the recommended items.

### 2.4 Summary and Research Questions

Building health-aware recommender systems raises two important challenges: 1) finding the healthy recipe that a specific user is the most likely to appreciate and 2) persuading the user to select that recipe. While the first challenge requires to optimize recommendation algorithms, the second one demands the adoption of specific designs.

Endowing recommender systems with task-oriented conversational skills is a good way to improve users’ experience during their interaction (Radlinski and Craswell, 2017). Moreover, systems building rapport with their users using social conversational skills can be perceived as more persuasive (Cassell and Bickmore, 2003; Pecune et al., 2019). Therefore, our first two research questions are:

**RQ1:** Does the possibility to interact with a food recommender system by typing answers influence users’ perception of the recommender system and users’ intention to cook recommended recipes?

**RQ2:** Do rapport-building conversational strategies influence users’ perception of a food recommender system and users’ intention to cook recommended recipes?

We learned from the literature on persuasive systems that adding alternatives that meet specific criteria could nudge people towards healthy eating (Fasolo et al., 2006; Lee et al., 2011; Elsweiler et al., 2017). However, none of the options presented to users in these works are matching user preferences. Hence, our fourth research question is:

**RQ3:** Are people more likely to accept healthy recipes matching their preferences when presented with alternative options?

The literature on recommender systems demonstrates how explanations influence people’s perception of recommended items (Symeonidis et al., 2009; Gedikli et al., 2014; Nunes and Jannach, 2017; Kunkel et al., 2019; Pecune et al., 2019). In the context of multiple recommendations, highlighting the trade-off between different alternatives offers a better support to users decision-making (Pu and Chen, 2007). However, food related experiments show that explicitly informing people about food healthiness might be counterproductive (Guthrie et al., 2015). Therefore, our fifth and last research question is:

**RQ4:** Are people more likely to accept healthy recommendations when explicitly told the recipe recommended to them is healthier than what they would usually eat?

## 3 Conversational Agent Architecture

**FIGURE 1:**
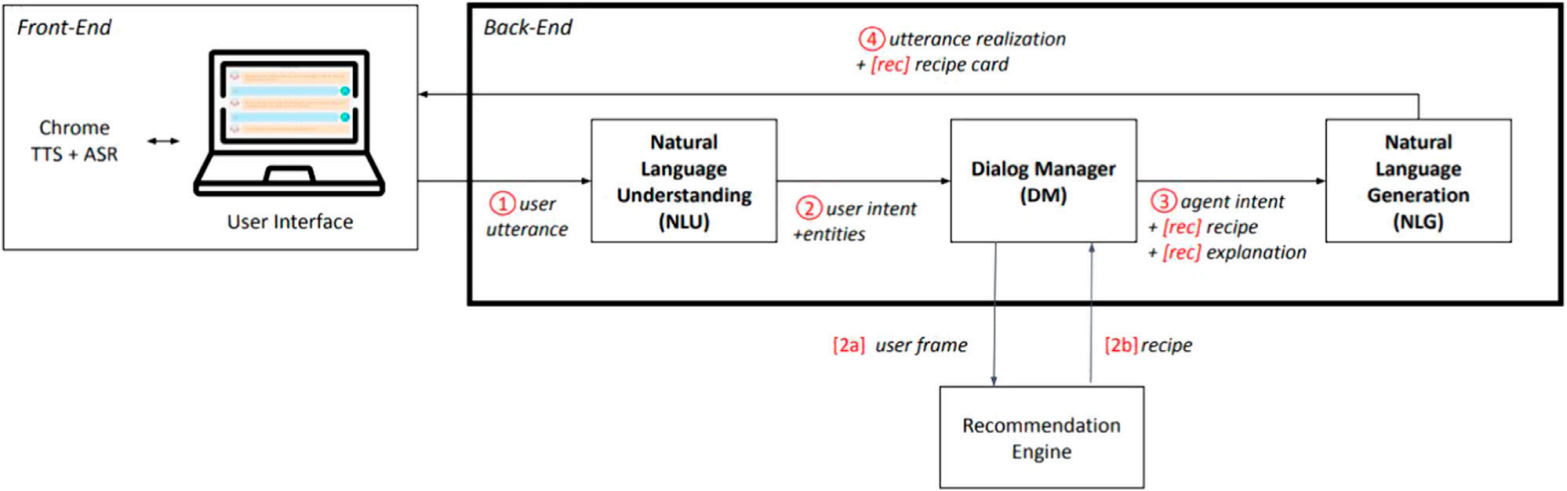
Architecture of Cora, our COnversational Recommender Agent, with recommendation-only items preceded by brackets.

To answer to our research questions, we built and deployed Cora, a conversational agent that recommends recipes to its users through a dialogue. The architecture of Cora is depicted in Figure 1 and the different components are described below.

### 3.1 Front-End

The front-end consists of a web page that allows each client to communicate with the server. Chat messages are displayed in a single scroll-down chat window. Whenever Cora recommends a recipe, a poster including the recipe’s picture, ingredients, and cooking steps is displayed in the conversation. Cora can display one, two, or three recommendations at the same time, depending on its Comparison-Mode. The user initiates the conversation by saying “Hello Cora”. To have better control over turn taking, the user cannot send a second message to Cora until it has answered the first one. We set up two different interfaces (User-Mode) for users to chat with Cora. In the *chat-mode*, the users write their messages to Cora by typing free text in a text input at the bottom of the chat window. In the *buttons-mode*, the users select pre-defined messages to send to Cora by clicking on buttons and/or selecting options in drop-down menus. For example, to the question “What do you think about this recipe?” users can answer either “I like it, thank you!”, “No, I don’t like the recipe”, “No, I don’t like #ingredient” or “No, other reason”. All the answer options were defined based on the most recurrent answers given by users in our prior pilot.

### 3.2 Back-End

The back-end consists of a server developed in *Python* which handles multiple simultaneous client connections and disconnections. For each new client, the server creates a dedicated Cora-agent. A Cora-agent is composed of three modules: 1) the Natural Language Understanding (NLU) module, in charge of making sense of what the user is saying, 2) a Dialog Manager (DM), deciding what to say next based on the output of the NLU and 3) a Natural Language Generation (NLG) module, generating sentences in natural language based on the output of the DM. Each module is described in more details below. The server is then in charge of distributing clients’ messages to the corresponding Cora-agents as well as Cora-agents’ messages to the corresponding clients.

#### 3.2.1 Natural Language Understanding

The first component triggered is the Natural Language Understanding (NLU) module, which extracts communicative intentions and entities from users’ utterances. Our NLU module uses the *Python* libraries nltk and Spacy to do lemmatization, dependency parsing and POS tagging on the utterance. We extract relevant entities by matching words/lemmas with a set of entities (i.e., ingredients, diets and intolerances) provided by the Spoonacular API^2^ that we use to recommend recipes. Given the entities, the POS tags and the dependency tags, the NLU module then uses a set of rules to determine the user’s intent, the associated entity and entity-type as well as a valence. For instance, the output corresponding to the input “I don’t like mushrooms” is {intent: ”inform”, entity_type: ”food”, entity: ”mushroom”, valence: ”-”}. This information is sent to the DM.

#### 3.2.2 Dialog Management

We designed our Dialog Manager (DM) as a finite state machine. It takes the user intent, entity-type, entity and valence extracted from the user’s utterance as inputs; it then uses these to transition to each new state based on the current state of the dialog and a set of rules. The DM stores the user’s recognized entities to keep track of their preferences in a *user frame*. The content of the user frame is used during the recommendation process.

We defined two interaction modes for Cora (Cora-Mode). In the *task-conv* mode, Cora focuses exclusively on its recommendation task. In the *social-conv* mode, Cora uses all the conversational strategies defined in 5.2 to build rapport with its users, including small-talk at the beginning of the conversation. We therefore defined a specific finite state machine for the *social-conv* to include additional states corresponding to the small-talk phase.

#### 3.2.3 Natural Language Generation

**FIGURE 2:**
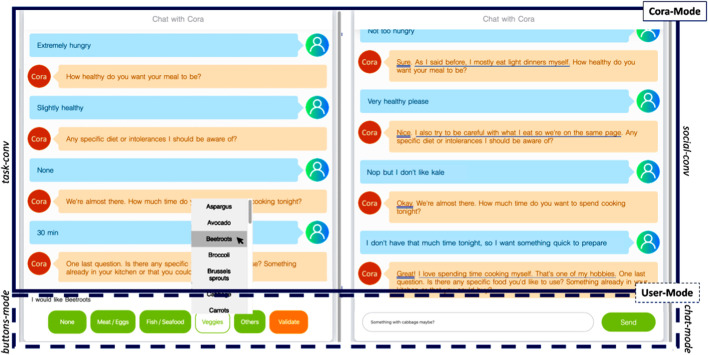
Excerpts of interactions between Cora and its user showing the two Cora-Modes (*task-conv* vs *social-conv*) and the two User-Modes (*buttons-mode* vs *chat-mode*) used in our experiment. The rapport-building conversational strategies used by Cora in the *social-conv* mode are underlined in the dialogue: acknowledgments with a double line, reciprocal appreciations with a single line and self-disclosures with a dotted line.

Given the user-utterance data outputted by the NLU, the dialog state outputted by the DM, Cora’s interaction mode (Cora-Mode), and Cora’s explanation mode (Expl-Mode), the Natural Language Generation (NLG) module uses a lookup table to generate an utterance in natural language. In the *social-conv* mode, the generated utterance contains one or more rapport-building strategies, which is not the case in the *task-conv* mode. Figure 2 presents two samples of interactions depicting the difference between the two Cora-Modes and User-Modes.

## 4 Experiments Overview

We used Cora in two different experiments whose goal was to explore the design of persuasive conversational systems for promoting healthy eating behaviors. Each of these experiments investigated specific persuasion techniques: rapport-building conversational strategies and nudging. Both experiments were approved by the local ethics committee of Glasgow University.

In the first experiment depicted in Section 5, we used Cora to investigate whether rapport-building food recommender systems would be perceived as more persuasive compared to task-oriented ones. In this experiment, we mostly focused on the design of the conversation leading to the recommendation of a recipe. For our first research question **RQ1**, we evaluated two different interaction modes (User-Mode): one in which users’ were able to chat freely with Cora (*chat-mode*), and another where users interacted with Cora using buttons and drop-down lists (*buttons-mode*). For our second research question **RQ2**, we tested two different conversational modes (Cora-Mode): a task-oriented mode (*task-conv*), and a social mode (*social-conv*) in which Cora used rapport-building conversational strategies during the interaction. We expected both (*chat-mode*) and (*social-conv*) versions of Cora to have a positive impact not only on rapport, but also on the perceived quality of the system and on users’ intention to cook recommended recipes.

In the second experiment described in Section 6, we built on the results of our first experiment to improve our system before investigating the impact of two types of nudges on Cora’s persuasion. In this experiment, we focused on the delivery phase of the recommendation process and explored different ways to present healthy recipes to the user. For our third research question **RQ3**, we evaluated three different comparison types for Cora’s recommendations (Comparison-Mode). Cora either recommended one single healthy recipe (*no-comp*), compared this healthy recipe with one matching users’ preferences (*pref-comp*), or compared these two recipes with an additional decoy (*decoy-comp*). For our fourth and last research question **RQ4**, we compared two different types of explanations (Expl-Mode): one where Cora relied on the recipes’ features to justify its recommendation (*feat-expl*), and another where Cora did not deliver any explanation (*no-expl*). We expected both (*decoy-comp*) and (*feat-expl*) versions of Cora to have a positive impact on the perceived quality of the system.

## 5 Experiment 1: Evaluating the Influence of Cora’s Conversational Skills and Interaction Mode

To answer our research questions **RQ1** and **RQ2**, we designed an experiment investigating how our system’s conversational skills (Cora-Mode) and interaction mode (User-Mode) influenced the perceived quality of both the system and the interaction, in addition to users’ intention to cook recommended recipes.

### 5.1 Recommendation Process

Cora’s main challenge is to convince users to select recipes that are healthier than what they would usually cook. To that end, Cora follows the traditional interview/delivery structure (Wärnestål et al., 2007; Jugovac and Jannach, 2017). During the interview phase, Cora first establishes users’ profiles based on their eating habits. Cora then gathers users’ constraints by asking specific questions about the recipe they would like to get. After that, Cora enters the delivery phase and recommends a recipe. Then, the user can accept or reject the recommendation. If the user refines his/her preferences (e.g., by saying he/she doesn’t like one of the ingredient of the recipe) the system updates its knowledge accordingly and recommends another recipe.

Work in grounded cognition has shown that people’s eating behaviors are driven by many mechanisms that they are most often not aware of (Chen et al., 2016; Papies et al., 2019). For example, different foods can be associated to different emotions. Each food can then be described in terms of the emotion it elicits in people, and assigned a global emotional score. Similarly, foods can be described in terms of *healthiness* and *fillingness* (how full does one feel after eating that food) and assigned fillingness and healthiness scores. As these two dimensions have been found to be good predictors for the frequency to which a food is eaten and for the acceptance rate of food recommender systems (Harvey et al., 2013), we rely on them to design our recommendation process in this experiment.

We first collected a Food database by regrouping the 40 ingredients that people most frequently cook and eat for dinner. Each ingredient of the database is characterized by healthiness and fillingness scores in [ − 1, 1] that were computed by averaging individual healthiness and fillingness scores assigned by hundreds of participants (e.g., salmon is associated with a healthiness value of 0.926 and a fillingness value of 0.678).

#### 5.1.1 Step 1: Collecting User Profile

The trait preferences corresponding to the user profile are gathered through a questionnaire that users answer prior to the interaction with Cora. Users are asked to rate how frequently they eat seven specific foods using 7-point Likert scales (anchors: 0 = never, 6 = once a day). These seven foods were selected from our healthiness-fillingness food database, after filtering out foods that are not compatible with a vegan diet (e.g., steak). Specifically, we selected the two items of our database with the highest ratings and the two items with the lowest ratings on the healthiness dimension and on the fillingness dimension, thus obtaining two sets of four items each. The two sets of selected food having one item in common, the final set has seven items.

To compute a trait healthiness preference score *p*(*t*)_*h*_ ∈ [ − 1, 1] for a user, we first calculate, for each food *j*, a healthiness score *s*_*h*,*j*_ as:

$$s_{h,j}=\left\{\begin{array}{c} freq_{j}\text{ if }j\text{ is a healthy food}\\ -freq_{j}\text{ if }j\text{ is not a healthy food,} \end{array}\right.$$

where *freq*_*j*_ is the frequency at which the user eats food *j*, as self-reported on our Likert-scale. We then sum those scores to obtain the user’s trait healthiness score *p*(*t*)_*h*_ = *∑*_*j*_ *s*_*h*,*j*_. We proceed in a similar way to calculate a trait fillingness preference score *p*_*t*_(*f*).

Even though this data is collected prior to the beginning of interaction, it is stored in the DM’s user frame and available during the interaction.

#### 5.1.2 Step 2: Gathering User’s Constraints

The state preferences representing users’ constraints are collected during the interaction with Cora and correspond to the answers to “How healthy do you want your meal to be?” for healthiness and “How hungry are you?” for fillingness. The NLU extracts from the user’s utterance a desired level of healthiness (resp. fillingness) converted to a value *p*(*s*)_*h*_ (resp. *p*(*s*)_*f*_) in { − 1, − .75, − .5 − .25, 0, .25, .5.75, 1}. For example, “no preference” will be mapped to a value of 0 while “slightly” will be mapped to a value of .25.

The user’s preferences are then averaged over trait and state values, resulting in two values *p*_*h*_ and *p*_*f*_ in [ − 1, 1].

Besides the healthiness and fillingness dimensions, the data collected during our pilot study showed that three other elements are critical when it comes to recommend a recipe: the diet/intolerances of the user (e.g., vegan or intolerant to gluten), the amount of time the user is willing to spend cooking, and whether there is a specific ingredient the user wants to use. Those preferences are also gathered during the interview phase of the dialog with Cora.

#### 5.1.3 Step 3: Delivering Recommendations

When the interview phase is over, Cora recommends recipes to the user. To do so, the DM goes through the following steps: 1) find an ingredient if the user did not specify one during the interview phase, 2) find a recipe with this ingredient and 3) use the user’s feedback for subsequent recommendations.

##### 5.1.3.1 Ingredient

If the user did not provide one, we want to select the best ingredient for the user to use in the recipe. Leveraging our Food Database, the DM generates a list of preferred ingredients *ingredients*_*list* for the user. To do so, the users’ preferences for healthiness and fillingness are represented as a vector $p=\left\langle p_{h},p_{f}\right\rangle$ . Each ingredient of the Food Database is represented in the same vector space, and ingredients are sorted by the distance of their vector to *p*, with the closest ingredient as the first one of the list. Ingredients that the user dislikes or cannot eat (e.g., if vegan) are excluded from the list.

##### 5.1.3.2 Recipe

To recommend a recipe to the user, the DM uses the Spoonacular API. This API allows us to query the Spoonacular database for recipes including or excluding a specific ingredient, that correspond to a specific diet (e.g., vegan) and/or take into account intolerances (e.g., to gluten) and that can be cooked in a specific amount of time. Each recipe received from the API is described by a title, a list of ingredients, a list of preparation steps (i.e., instructions), and some nutritional information, including a global healthiness score and an amount of calories that we map to our fillingness score. The DM queries the API for two recipes containing the first ingredient of the *ingredients*_*list* and stores the results in a *recipe*_*list*. Similarly to ingredients, recipes of the *recipe*_*list* are represented in the healthiness-fillingness vector space and are sorted by distance to the preference vector *p*. If the DM receives less than two results from the API or if at any point the *recipe*_*list* is empty, the first ingredient in *ingredients*_*list* is popped out of the list and the DM queries the API for two more recipes following the same procedure.

##### 5.1.3.3 Using user feedback

If Cora has to give more than one recommendation, it uses the user’s feedback to select another recipe: 1) If the user answered they don’t like an ingredient (resp. recipe), the disliked ingredient is stored in a *disliked*_*ingredients*_*list* (resp. *disliked*_*recipes*_*list*). The DM then removes from *recipe*_*list* all recipes that contain disliked ingredients, as well as recipes with a title similar to disliked recipes. We do fuzzy matching to compute the distance between two titles, using Levenshtein distance. If *recipe*_*list* is empty, the DM goes back to step 2.

### 5.2 Rapport-Building Dialogue

We rely on the computational model presented in (Zhao et al., 2014) to determine a list of conversational strategies for Cora to use during the interaction (see Figure 2). The computational model describes how and when specific conversational strategies are used to build, maintain, or destroy rapport during an interaction. More specifically, we implemented the following strategies:

#### 5.2.1 Small Talk as an Introductory Phase

Small talk can be seen as a way to break the ice during a first interaction with someone (Svennevig, 2000). Although questions like “how are you?” can result in very deep answers (Coupland et al., 1992), small talk usually consists of safe and non-intimate questions. Previous work in the domain of conversational agents emphasized the role of small talk in task oriented contexts (Cassell and Bickmore, 2003). In our work, small-talk consists of Cora asking four questions at the beginning of the interaction before reaching the preference gathering phase. Cora first asks about users’ name and whether they are doing alright. Then, Cora asks about what the users usually eat for dinner, and why they eat such food.

#### 5.2.2 Self-Disclosures

A self-disclosure is the communication process of revealing information about oneself to somebody else (Greene et al., 2006). Previous work already emphasized the need to endow conversational systems with the ability to self-disclose personal information (Lee and Choi, 2017; Tada et al., 2018). The study depicted in (Bickmore et al., 2009) compares two different agents: the first agent discloses a human-like story as its own, while the second one discloses information about a human he knows. People enjoyed significantly more the conversations with the first agent, and did not find such agent as more dishonest, despite the fact that the story disclosed by the first agent was not plausible. This result is corroborated in (Gilani et al., 2016). Cora thus discloses information about itself to its users during the small-talk and preference gathering phases. Some examples of self-disclosures are:“I love spending time cooking myself, that’s one of my hobbies.”, “I try to be careful with what I eat.”, or “I mostly eat light dinners.”

#### 5.2.3 Feedback and Acknowledgements

Acknowledgements are a way to show understanding of a previous utterance during a conversation (Traum and Hinkelman, 1992). In this work, Cora uses such acknowledgements (e.g., “okay,” “right,” “sure”, etc … ) to show that it understood what the user just said. Cora also uses reciprocal appreciation to give feedback to what users said and build rapport with them at the same time. As explained by (Heider, 2013), people tend to appreciate their interlocutor more when they express similar attitudes toward an opinion, an object, or another person. For instance, if one user says that he/she is hungry, Cora gives a feedback saying “I’m hungry too!”

#### 5.2.4 Personal Opinions as Explanations

As shown in (Pecune et al., 2019), a system justifying its recommendation using its “own” personal opinion or personal experience can increase rapport during an interaction. In this work, Cora uses explanations such as “It’s personally one of my favorites!” or “This recipe looks delicious.” whenever it recommends a recipe to its user.

### 5.3 Stimuli

We identified two different independent variables, corresponding to the DM/NLG modes and the front-end interfaces described in Section 3. The first one represents Cora’s conversational mode (Cora-Mode) as a between-subject independent variable with two levels: socially-oriented conversation (*social-conv*) in which Cora liven up the conversation by using rapport-building strategies as described in 5.2, and task-oriented conversation (*task-conv*) in which Cora simply asks questions and delivers recommendations. The second between-subject variable represents the way the user can interact with Cora (User-Mode) and has two levels: a button mode (*buttons-mode*) in which users interact with Cora using buttons and drop-down lists only, and a chat mode (*chat-mode*) in which users can type whatever they want in natural language.

Our experiment has a 2x2 design with Cora-Mode and User-Mode as between-subject variables. Two samples of interactions depicting the difference between the conditions are presented in Figure 2. In each of the four conditions, participants followed the same procedure. They were first presented with a consent form informing them about the procedure of the experiment. Those who agreed to partake in the study were then presented with a short description of the task explaining the context of the interaction (i.e., the participant has to find a recipe to cook for tonight). Before interacting with Cora, participants had to fill in a questionnaire about their eating habits as described in Section 5.1.1. Each participant was then randomly assigned to a group according to the different independent variables and interacted with Cora following the recommendation scenario described in Section 5.1. After the end of their interaction, participants took three surveys that measured the quality of the interaction, the quality of the system and their intention to cook the recipes recommended to them. In addition to these three surveys, two open-ended questions asked participants their thoughts about Cora and about the experiment. Finally, participants answered a demographics questionnaire.

### 5.4 Measurements

We measured the following three constructs in our experiment. 1) We relied on rapport (Tickle-Degnen and Rosenthal, 1990) -a notion commonly used in the domain of human-agent interactions (Gratch et al., 2006; Zhao et al., 2018a)- as a proxy to measure the quality of the interaction. The eight different items we used to measure rapport are listed in Table 1. 2) The perceived quality of the conversational recommender system was measured using a questionnaire derived from (Pu et al., 2011). For participants’ beliefs about the system, we measured the system’s perceived usefulness, ease of use, transparency, accuracy, and user control. Regarding participants’ attitude, we measured their overall satisfaction and how much they trust the system. To measure participants’ behavioral intentions, we asked participants whether they would use Cora in the future. The seven different items we used encompass multiple aspects of a recommender system’s task performance and are listed in Table 2. We also added an eighth item to assess the perceived healthiness of the recipes recommended. 3) Finally, we measured participants’ intention the cook the recommended recipes through a questionnaire adapted from (Conner et al., 2002). The five items we used to measure intention to cook are listed in Table 3. All answers for the three questionnaires were on a 7-point Likert scale (anchors: 1 = completely disagree, 7 = completely agree).

**TABLE 1:** Subjective questionnaire adapted from (Zhao et al., 2018a) to measure users’ perceived quality of the interaction.

Dimensions	Subjective items
Coordination	I felt I was in sync with Cora
	I was able to say everything I wanted to say during the interaction
Mutual Attentiveness	Cora was interested in what I was saying
	Cora was respectful to me and considered to my concerns
Positivity	Cora was warm and caring
	Cora was friendly to me
Rapport	Cora and I established rapport
	I felt I had no connection with Cora

**TABLE 2:** Subjective questionnaire adapted from (Pu et al., 2011) to measure users’ perceived quality of the system.

Dimensions	Subjective items
Accuracy	The recipes recommended to me during this interaction matched my preferences
User Control	Cora allowed me to specify and change my preferences during the interaction
Intention to Return	I would use Cora to get recipe recommendations in the future
Ease of Use	I easily found the recipes I was looking for
Healthiness	The recipes recommended by Cora were healthy
Satisfaction	I was satisfied with the recipes recommended to me
Usefulness	Cora provided sufficient details about the recipes recommended
Transparency	Cora explained her reasoning behind the recommendations

**TABLE 3:** Subjective questionnaire adapted from (Conner et al., 2002) to measure users’ intention to cook.

Dimensions	Subjective items
Intention to Cook	I want to make the recipe recommended to me
	I expect to make the recipe recommended to me
	It is likely I will make the recipe recommended to me
	I intend to make the recipe recommended to me
	I will try to make the recipe recommended to me

### 5.5 Hypotheses

We learned from previous work that rapport-building conversational strategies increase users’ satisfaction (Lee and Choi, 2017; Pecune et al., 2019) and users’ intention to use the system (Lee and Choi, 2017). Hence, we expect our system’s conversational mode (Cora-Mode) to have an impact on the perceived quality of the system and on user’s intention to cook. We hypothesize the following:

**H1-a**:The interactions with a system that engages participants using rapport-building conversational strategies (*social-conv*) will be perceived as better than the interactions with a system that engages participants through a task-oriented conversation (*task-conv*).

**H1-b**: The quality of a system that engages participants using rapport-building conversational strategies (*social-conv*) will be perceived as higher than the quality of a system that engages participants through a task-oriented conversation (*task-conv*).

**H1-c**: Participants interacting with a system engaging them using rapport-building conversational strategies (*social-conv*) will be more likely to report they want to cook one of the recommended recipes compared to participants interacting with a system engaging them through a task-oriented conversation (*task-conv*).

Building rapport is a dyadic process, which would require the user to reciprocate the use of conversational strategies during the interaction (Zhao et al., 2014). Thus, we expect users’ interaction mode (User-Mode) to have an impact on users’ overall experience and intention to cook. We hypothesize the following:

**H2-a**: Participants chatting with a system (*chat-mode*) will perceive the interaction as better compared to participants interacting with a system using buttons and drop-down menus (*buttons-mode*).

**H2-b**: Participants chatting with a system (*chat-mode*) will perceive its quality as higher compared to participants interacting with a system using buttons and drop-down menus (*buttons-mode*).

**H2-c**:Participants chatting with a system (*chat-mode*) will be more likely to report they want to cook one of the recommended recipes compared to participants interacting with a system using buttons and drop-down menus (*buttons-mode*).

### 5.6 Results

We collected 106 interactions on Amazon Mechanical Turk (*N* = 106), with a balanced number of interactions per condition. To ensure the quality of the data collected, all participants had at least a 95% approval rating with more than 100 previous HITs validated. Participants were paid USD 0.75 and spent an average of 6 min and 11 s (std = 3 min59 s) on the task. Participants were mostly men (67%), from the United States (67%), working full-time (80%), with a degree of higher education (89%). They were aged 18–29 (24%), 30–49 (65%) or 50–69 (11%). All participants cook at least occasionally and most of them cook at least once a day (43%) or several times a week (44%). Most participants said they are very familiar (55%) or somewhat familiar (41%) with conversational assistants.

Our conversational agent recommended 238 recipes in total, with an average of 2.24 recommendations per interaction (std = 1.69). The average acceptance rate for the recommendations was 0.79 (std = 0.39).

#### 5.6.1 Quality of the Interaction

We conducted a 2x2 factorial MANOVA (i.e., multivariate analysis of variance) with Cora-Mode and User-Mode as between-subject factors. The dependent measures were the eight questions presented in Table 1. The factorial MANOVA revealed a significant main effect of Cora-Mode (F (1, 102) = 5.088; p < .05) on the perceived quality of the interaction. There was no main effect of User-Mode (F (1, 102) = 1.214; *p* = .27) on the perceived quality of the interaction and the interaction between the two variables was not significant (F (1; 102) = 3.453; *p* = .06). H1-a is validated, but not H2-a.

For our follow-up analysis, we looked at univariate effects of Cora-Mode on each dependent measure with Student’s t-tests. Our results showed a significant main effect of Cora-Mode on the item “Cora and I established rapport” (F (1, 104) = 16.24; p < .001; *η* ^2^ = .14). This result shows that the version of Cora engaging the participants using conversational strategies was effectively able to build rapport with them. For all the questionnaire items, the system was rated with higher scores when engaging participants in a social dialogue (*social-conv*) compared to a task dialogue (*task-conv*). The quality of the interaction was rated high across all conditions (mean and std for the quality of the interaction across all conditions m = 4.58, std = 1.04). We report and compare the summary of all means for the eight dependent variables in Supplementary Figure S1 and Supplementary Figure S2.

#### 5.6.2 Quality of the System

We conducted a 2x2 factorial MANOVA with Cora-Mode and User-Mode as between-subject factors. The dependent measures were the eight questions presented in Table 2. The factorial MANOVA revealed a significant main effect of Cora-Mode (F (1, 102) = 4.325; p < .05) on the perceived quality of the system. There was no main effect of User-Mode (F (1, 102) = 2.354; *p* = .13) on the perceived quality of the system and the interaction between the two variables was not significant (F (1; 102) = 1.167; *p* = .28). H1-b is validated, but not H2-b.

Similar to the previous section, we performed a follow-up analysis that looked at univariate effects of Cora Mode on each dependent measure with Student’s t-tests. Our results showed a significant main effect of Cora-Mode on the participants Intention to Return (F (1, 104) = 5.77; p < .05; *η* ^2^ = .05) and Perceived Usefulness (F (1, 104) = 4.00; p < .05; *η* ^2^ = .04). Participants are more willing to use a system able to engage them in a rapport-building conversation, and they also perceive that a rapport-building system delivers more details about the recommendations. For all the questionnaire items, the system was rated with higher scores when engaging participants in a social dialogue (*social-conv*) compared to a task dialogue (*task-conv*). The quality of the system was rated high across all conditions (mean and std for the quality of the system across all conditions m = 4.50; std = 1.16). We report and compare the summary of all means for the eight dependent variables in Supplementary Figure S3 and Supplementary Figure S4.

#### 5.6.3 Intention to Cook

We conducted a 2x2 factorial MANOVA with Cora-Mode and User-Mode as between-subject factors. The dependent measures were the five questions presented in Table 3. The factorial MANOVA revealed no significant main effect of Cora-Mode (F (1, 102) = 2.660; *p* = .1) and User-Mode (F (1, 102) = 2.852; *p* = .09) on the perceived quality of the system. The interaction between the two variables was not significant (F (1; 102) = 2.233; *p* = .14). H1-c and H2-c are not validated. For all the questionnaire items, the system was rated with higher scores when engaging participants in a social dialogue (*social-conv*) compared to a task dialogue (*task-conv*). The scores obtained by the *button-mode* version were also higher than the scores obtained by the *chat-mode* version for all the items. The intention to cook was rated high across all conditions (mean and std for intention to cook across all conditions m = 4.41; std = 1.39). We report and compare the summary of all means for the five dependent variables in Supplementary Figure S5 and Supplementary Figure S6.

### 5.7 Discussion

The ratings above the average obtained across each condition combined with the high acceptance rate show that participants were generally satisfied with Cora and its recommendations. Regardless of the quality of the recommendations, it seems that endowing recommender systems with rapport-building abilities has a positive influence on users’ perception. That is corroborated by the fact that the rapport-building version of Cora systematically obtained better scores than its task-oriented counterpart, and lower standard deviations. In other words, participants preferred the rapport-building version of Cora, and their ratings of this version were more consistent. Furthermore, not only are participants significantly more willing to use a system able to engage them in a rapport-building conversation, but they also perceive that a rapport-building system delivers significantly more details about the recommendations, although it simply gives its “own” personal opinion. The latter result is consistent with (Pecune et al., 2019), in which recommender systems able to talk about their opinions and experiences were also perceived as significantly more useful compared to systems using feature-based explanations or no explanation at all. This highlights the importance of endowing recommender systems with the ability to express their own opinions about the items they recommend.

Although we expected rapport-building conversational strategies, especially acknowledgements, to have a positive influence on mutual attentiveness, the differences were not significant. This can be due to the type of acknowledgement Cora used during the interaction. We decided to use short neutral acknowledgements such as “okay”, “great”, “alright” to show Cora’s understanding during the interaction. However, it might have been better for Cora to repeat part of the user’s sentence to implicitly confirm its understanding, as it is done in (Bohus and Rudnicky, 2005). The absence of significant results regarding the positivity can be explained by the absence of praise and negative self-disclosures in the interaction. According to (Zhao et al., 2014), these two strategies are quite important to maintain people’s face and to appear more friendly.

**FIGURE 3:**
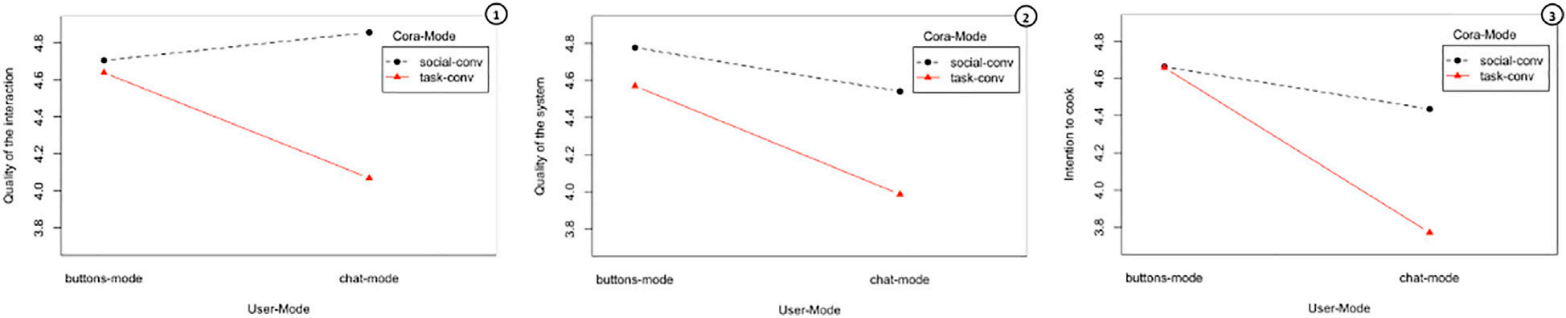
Interaction graphs between Cora-Mode and User-Mode regarding the average scores for all the items related to the quality of the interaction, the quality of the system and users intention to cook.

Contrary to our initial intuition, we found that the chat version of Cora generally obtained lower scores compared to its button counterpart. However, if we look at the interaction graphs in Figure 3, we notice two interesting results. First, participants who interacted with a rapport-building Cora via free text rated the quality of the interaction higher than the ones who interacted with the rapport-building Cora via buttons and drop-down menus. This shows that allowing users to freely reciprocate a recommender system’s conversational strategies during the interaction helps to improve their overall experience. Participants who interacted with the rapport-building Cora via free text found the system *“fun and engaging”* and thought Cora *“sounded so sympathetic and gave off a vibe of someone who cares about people’s opinions”*. Second, the difference in the ratings of the quality of interaction and intention to cook between button-mode and chat-mode was lower when Cora was using rapport-building conversational strategies. Rapport-building strategies seem to have mitigated the issues related to natural language understanding. Participants were more forgiving towards the system when it was using rapport-building strategies, as hinted by one comment: *“Cora kind of ignored my dietary preferences, but she sounded pretty natural compared to most chat bots”*.

We found a potential explanation for the lower scores of chat-mode Cora by looking at the interactions logs. Indeed, some specific sentences written by users in the chat-mode were not correctly understood by Cora. We identified two categories of errors: 1) sentences that were misclassified by our natural language understanding components leading to an inaccurate recommendation in the end and 2) users request that were not handled by our system, hence not classified at all, leading to users’ frustration. Both categories had a negative impact on users’ rating of system quality and intention to cook.

Furthermore, we found that people who were not familiar with conversational assistants rated the chat-mode version of Cora significantly lower compared to the button-mode. These participants might have been oblivious of the current conversational assistant limitations or conventions and preferred more conventional interfaces that they found more reliable. Although we expected the button-mode to be too limiting, only one participant commented that *“some answers felt restrictive and made me feel like I wouldn’t be able to say what I meant with the ready-made answers.”*

One comment from a participant who interacted with rapport-building Cora shed light on a very interesting point: *“It was good, but there’s too much unimportant conversation in my opinion. There should be two modes: talkative and time-efficient”*. This is consistent with the work presented in (Jain et al., 2018) in which the authors classified the users of a recommender system in two categories: those who actively wanted to build rapport with the recommender system, and those who wanted to get a recommendation in the most efficient way. Since our results show that the rapport building system obtained better scores than its task-oriented counterpart, we can hypothesize that the negative impact induced by building rapport with a person who only focuses on the task is smaller than the positive impact induced by building rapport with a person who cares about it. However, building systems able to identify the type of users they are interacting with and to adapt their strategies could improve overall users’ experience.

The recommendation process we relied on during this experiment had some limitations. Although the ingredients in our database were associated with values representing perceived healthiness and perceived fillingness, people are not always able to judge accurately the healthiness of specific food items (Trattner and Elsweiler, 2017a). Hence, the perceived healthiness of an ingredient might actually be very different from its real nutrition value, which might lead to an inaccurate recommendation. Another issue with our approach is that we did not consider the amount of a specific ingredient in a recipe. Although one single potato can be considered rather healthy, a recipe containing five of them might not be that healthy in the end.

## 6 Experiment 2: Evaluating the Influence of Cora’s Nudges and Explanations

To answer to our research questions **RQ3** and **RQ4**, we designed a second experiment investigating how recommendations explanations and recipes comparisons influence users’ perception of Cora. Before running the experiment, we built on the results discussed in Section 5.7 to update the design of Cora. To avoid any potential issue with our system’s NLU, we relied on pre-defined messages sent to Cora by clicking on buttons and/or selecting options in drop-down menus. Since the social version of Cora (*social-conv*) obtained better results compared to the plain task-oriented one, Cora used rapport-building strategies during the interaction.

### 6.1 Recommendation Process

To overcome the limitations of our recommendation process addressed in Section 5.7, we built our own recommendation engine. For this experiment, we scraped our recipe dataset from allrecipes.com, one of the most popular recipe websites in terms of traffic. Each recipe in the dataset is associated with a title, a picture, a list of ingredients, preparation steps, preparation and cooking times, number of servings, nutritional values, and ratings data. We also relied on (Trattner and Elsweiler, 2017b; Elsweiler et al., 2017) to calculate a *health*_*score* for each recipe based on its nutritional values. This health score ranges from 4 (healthiest) to 12 (least healthy).

**FIGURE 4:**
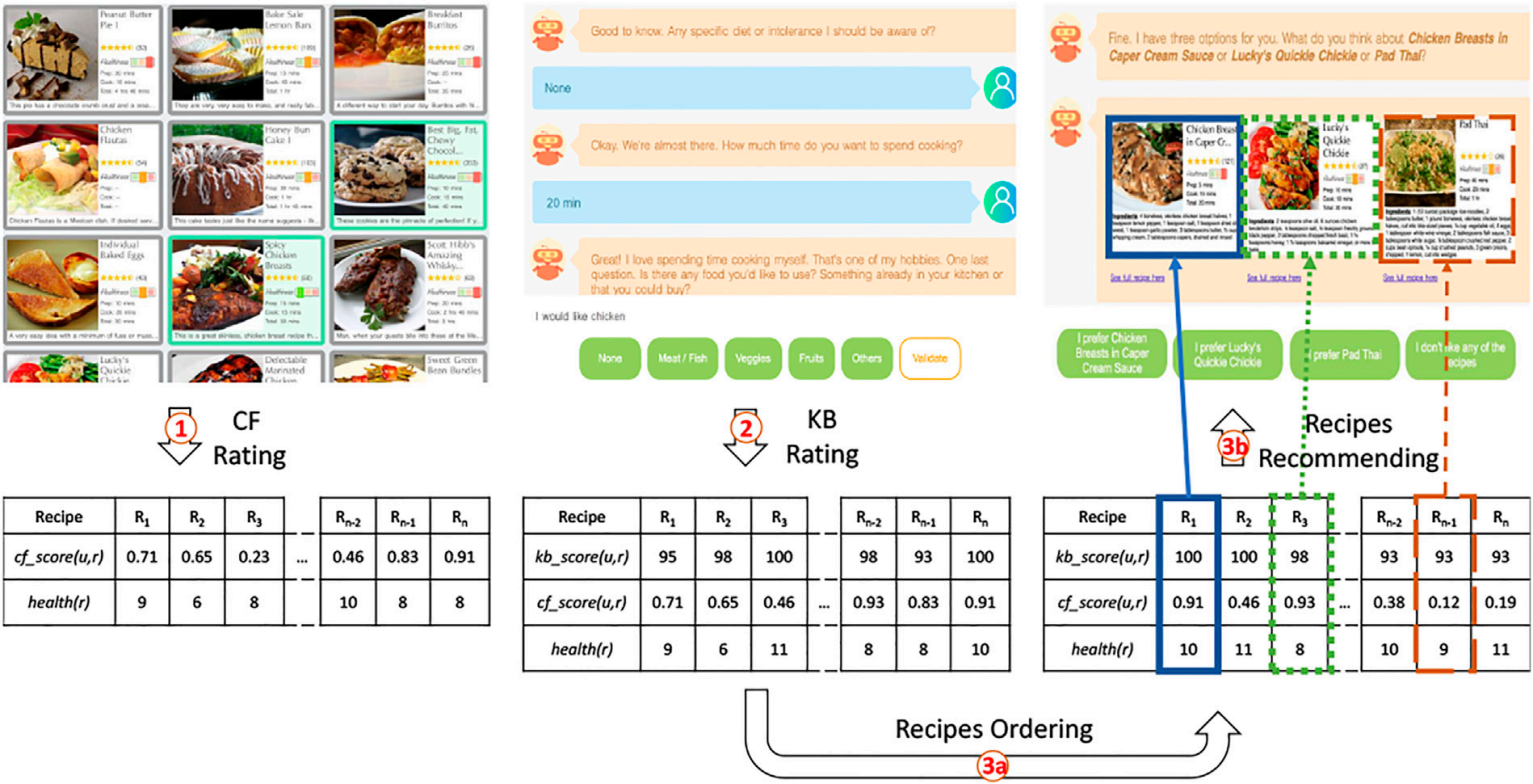
Overview of the recommendation Process. A user *u* first selects recipes he likes from a list of recipes. The system relies on this user profile to compute a *cf*_*score* (*u*, *r*) for each recipe *r* as explained in Section 6.1.1. The system then asks about user’s *u* specific constraints and computes a *kb*_*score* (*u*, *r*) as explained in Section 6.1.2. The system then orders the recipes according to their scores before delivering a recommendation to the user.

As in (Burke, 2002; Pecune et al., 2020a), Cora relies on a hybrid approach combining knowledge-based (KB) and collaborative filtering techniques (CF) for this experiment. The recommendation process has three steps. 1) First, the system relies on CF to rate all the recipes in the dataset based on the recipes the user likes. The predicted rating for a recipe *r* given a user *u* is noted *cf*_*score* (*u*, *r*). 2) In addition to *cf*_*score* (*u*, *r*), the system also computes a relevance score *kb*_*score* (*u*, *r*) for each recipe *r* based on user *u*’s constraints. 3) The system finally ranks the recipes given their *cf*_*score* (*u*, *r*) and *kb*_*score* (*u*, *r*) before delivering three different recommendations: a *pref*_*recipe*, a *healthy*_*recipe* and a *decoy*_*recipe*. The recommendation process is illustrated in Figure 4 and each step is detailed below.

#### 6.1.1 Step 1: Collecting User Profile

The first step of our recommendation process is to predict how much a user *u* will like each recipe *r* from our dataset given a list of recipes the same user *u* already likes, i.e., to compute the *cf*_*score* (*u*, *r*). To do so, we tested three different CF algorithms (Alternating Least Squares (ALS) (Takács and Tikk, 2012), Bayesian Personalized Ranking (BPR) (Rendle et al., 2009) and Logistic Matrix Factorization (LMF) (Johnson, 2014)) and compared their performance in terms of AUC (Schröder et al., 2011). To reduce data sparsity issues, we limited ourselves to recipes that had been reviewed by at least 25 users and users who had rated at least 30 recipes. Thus, our dense dataset contains 1,169 recipes and 1,339 users for a total of 70,945 ratings. We transformed existing users ratings in our dataset into confidence levels following the approach described in (Hu et al., 2008) before splitting our dataset into train, cross-validation and test sets. The best performing algorithm was ALS (average AUC = 0.694 over 100 train-test iterations), with a performance comparable to the one reported in (Trattner and Elsweiler, 2019) on a similar dataset. Our recommender system therefore relies on this algorithm to output, for a given user *u*, and for each recipe *r* a rating *cf*_*score* (*u*, *r*) ∈ [ − 1, 1].

#### 6.1.2 Step 2: Gathering User’s Constraints

The objective of the second phase is to take into account users’ current goals or constraints. To do so, the system rates each recipe *r* in the dataset based on user *u*’s constraints, i.e., compute the *kb*_*score* (*u*, *r*). In this work, we consider the three following constraints: the ingredients the user *u* wants, any specific diet the user *u* follows, and the maximum amount of time the user *u* wants to spend cooking. For each recipe *r*, the system checks whether user’s *u* constraints are satisfied. If that is the case (i.e., if the recipe contains all the ingredients, and corresponds to the user’s diet and cooking time), the recipe obtains the maximum *kb*_*score* (*u*, *r*) of 100. For each constraint that is not satisfied, the *kb*_*score* (*u*, *r*) is decreased by the values listed in Table 4.

**TABLE 4:** Penalties applied to *kb*_*score* (*u*, *r*) depending on the constraint relieved.

Constraint	Penalty applied to *kb*_*score* (*u*, *r*)
Diet	−10
Ingredient	−5
Time + at most 30 min	−2
Time + at least 30 min	−4

#### 6.1.3 Step 3: Delivering Recommendations

Once each recipe of the dataset is associated with a *cf*_*score* (*u*, *r*) and a *kb*_*score* (*u*, *r*), our system can finally deliver its recommendations. Recipes are first ranked based on their relevance score *kb*_*score* (*u*, *r*). Recipes with identical relevance scores *kb*_*score* (*u*, *r*) are then re-ordered based on their CF ratings *cf*_*score* (*u*, *r*). To investigate our second research question **RQ3**, our system can deliver up to three recommendations. The top-item *pref*_*recipe* of the list corresponds to the recipe best matching users’ preferences. To find the *healthy*_*recipe* we ideally want users to select, the system goes down the list to find the first recipe with a *health*_*score* lower than *pref*_*recipe*’s *health*_*score*. According to the decoy effect theory, the *decoy*_*recipe* should be less attractive than the *healthy*_*recipe* in all dimensions. Hence, the system selects a *decoy*_*recipe* with a lower *kb*_*score* (*u*, *r*) than the *healthy*_*recipe* as well as a less healthy *health*_*score*. These three recommendations are in line with the ones presented in (Fasolo et al., 2006) and described in Section 2.2. The *healthy*_*recipe* represents the target T (high *health*_*score*, medium similarity with the user’s preferences), the competitor C is *pref*_*recipe* (medium *health*_*score*, high similarity with the user’s preferences) and the decoy D is *decoy*_*recipe* (medium *health*_*score*, low similarity with the user’s preferences).

To investigate our research question **RQ4**, our system generates explanations to try to convince the user to choose a recipe slightly healthier than the one best matching their preferences. The form of the explanation generated depends on the number of recipes recommended: when recommending more than one recipe, the *healthy*_*recipe* is compared to the *pref*_*recipe* and presented as healthier. If only one recipe is recommended, the generated explanation differs depending on whether or not the recipe fully satisfies the user’s constraints. If that is the case (i.e., *kb*_*score* (*u*, *r*) = 100), the recipe is presented as the healthiest corresponding to the user’s preferences; otherwise the system specifies which preference is not satisfied and presents the recipe as healthier than other options. Examples of explanations for each case are given in Table 5.

**TABLE 5:** Explanations generated depending on the number of recipes recommended and on whether the user’s constraints were satisfied or not.

kb_scores of *healthy*_*recipe*	Recommended recipes	Explanation example
= 100	healthy_recipe	Chicken Katsu *is the healthiest recipe matching your preferences*
<100	healthy_recipe	Chicken Katsu *corresponds to your preferences except that it does not contain steak; but it is healthier than other options (contains less fat, sugar or salt)*
≤100	pref_recipe and healthy_recipe	Pan-Fried Steak with Marsala Sauce *best matches your preferences but* A Good Easy Garlic Chicken *is healthier as it contains less fat and sugars*
≤100	pref_recipe, healthy_recipe and decoy_recipe	Pan-Fried Steak with Marsala Sauce *best matches your preferences but* Creamy Broccoli Soup *is healthier*. *Asparagus* Parmesan *is also healthier but it does not contain chicken*

### 6.2 Stimuli

We identified two different independent variables. The first one represents Cora’s explanation mode (Expl-Mode) as a between-subject independent variable with two levels. In the (*feat-expl*) condition, Cora delivers feature-based explanations to justify its choice as explained in Section 6.1.3. Cora does not deliver any explanation in the (*no-expl*) condition. The second between-subject variable represents how many recipes users are getting (Comparison-Mode) and has three levels. The system only recommends the *healthy*_*recipe* in the (*no-comp*) condition. Users in the (*pref-comp*) condition are presented with both *healthy*_*recipe* and *pref*_*recipe*. In addition to the *healthy*_*recipe* and *pref*_*recipe*, Cora also recommends a *decoy*_*recipe* in the (*decoy-comp*) condition.

Our experiment has a 2x3 design with Expl-Mode and Comparison-Mode as between-subject variables. In each of the six conditions, participants followed the same procedure. They were first presented with a consent form informing them about the procedure of the experiment. Those who agreed to partake in the study were then presented with a short description of the task explaining the context of the interaction (i.e., the participant will be presented with a recipe recommendation). Each participant was then randomly assigned to a group according to the different independent variables and interacted with Cora following the traditional interview/delivery structure (Jugovac and Jannach, 2017). Cora first greeted the user and introduced itself before gathering the relevant pieces of information needed to recommend personalized and healthy recipe recommendations. To get a list of users’ preferred recipes, Cora asked them to select at least five recipes that they like amongst a grid of thirty recipes randomly selected from our dataset. This list of recipes was used to compute *cf*_*score* (*u*, *r*) as explained in Section 6.1.1. Cora then asked whether users were on a specific diet, whether users want to use a specific ingredient for their recipe, and how much time users are willing to spend to cook dinner. These constraints were used to compute *kb*_*score* (*u*, *r*) as explained in Section 6.1.2. After that, Cora entered the delivery phase and recommended recipes. The recommendations were presented to users in the form of recipe cards including a title, a picture, a list of ingredients, preparation and cooking times, the number of ratings and the average rating score (see Figure 4). The health score was displayed following the green (healthy), orange (moderately healthy) and red (unhealthy) traffic-light system provided by the Food Standard Agency (FSA, United Kingdom). The users were asked to either select one of the recipes recommended or reject the recommendations. After the end of their interaction, participants took a survey that measured the perceived quality of the system. In addition to this survey, two open-ended questions asked participants their thoughts about Cora and about the experiment. Finally, participants answered a demographics questionnaire.

We collected 289 interactions on the Prolific platform (*N* = 289), with a balanced number of interactions per condition. To ensure the quality of the data collected, all participants had at least a 95% approval rate with more than 100 previous assignments validated. Participants were paid £0.95 and spent an average of 7 min and 10 s (std = 3 min33 s) on the task.

### 6.3 Measurements

To evaluate the impact of our nudges on participants’ decision making, we measured whether they picked the *healthy*_*recipe* whenever it was recommended. In addition to that objective measure, we also evaluated the perceived quality of the conversational recommender system using a questionnaire similar to the one we used during our first experiment. For participants’ beliefs about the system, we measured the system’s perceived usefulness, ease of use, transparency, and accuracy. In this experiment, users were not able to change their preferences after Cora delivered the recommendations. Hence, we did not include the item measuring user control. Regarding participants’ attitude, we measured their overall satisfaction and how much they trust the system. Finally, to measure participants’ behavioral intentions, we asked participants whether they would cook the recipes they were recommended, and whether they would use Cora in the future. All answers for the questionnaire described in Table 6 were on a 7-point Likert scale (anchors: 1 = completely disagree, 7 = completely agree).

**TABLE 6:** Subjective questionnaire adapted from (Pu et al., 2011) to measure users’ perceived quality of the system.

Dimensions	Subjective items
Decision Confidence	The recipes recommended to me during this interaction matched my preferences
Intention to Return	I would use Cora to get recipe recommendations in the future
Perceived Effort	I easily found the recipes I was looking for
Recommendation Quality	I was satisfied with the recipes recommended to me
Perceived Usefulness	Cora provided sufficient details about the recipes recommended
Transparency	Cora explained her reasoning behind the recommendations
Trust	I am convinced of the recipes recommended to me
Intention to cook	I would cook the recipe recommended, given the opportunity

### 6.4 Hypotheses

We learned from previous work that recommendation explanations have a positive impact on users’ perception of a recommender system (Symeonidis et al., 2009; Gedikli et al., 2014; Nunes and Jannach, 2017; Pecune et al., 2019). Hence, we expect to find a similar positive impact of explanations on the perceived quality of our system and on user’s recipe selection. We hypothesize that the system’s explanation mode (Expl-Mode) will have a main effect on the perceived quality of the recommender system, and on whether participants select the *healthy*_*recipe* when given the choice.

**H1-a**: Participants interacting with a system that explains its recommendations (*yes-expl*) will be more likely to select the *healthy*_*recipe* compared to participants interacting with a system that recommend recipes without explanation (*no-expl*).

**H1-b**: The quality of a system that explains its recommendations (*yes-expl*) will be perceived as higher than the quality of a system that recommend recipes without explanation (*no-expl*).

Several experiments demonstrated that the decoy effect is an effective way to nudge people toward a specific choice (Fasolo et al., 2006; Lee et al., 2011). Therefore, we expect that comparing the *healthy*_*recipe* with a less attractive recipe could nudge people towards healthy eating. We hypothesize that users’ comparison mode (Comparison-Mode) will have a main effect on the participants’ likelihood to select the *healthy*_*recipe* when given the choice, and on the perceived quality of the conversational recommender system.

**H2-a**: Participants presented with a *decoy*_*recipe* (*decoy-comp*) will be more likely to accept the *healthy*_*recipe* compared to participants interacting with a system delivering only a *healthy*_*recipe* (*no-comp*) or a *healthy*_*recipe* next to the top matching recipe (*pref-comp*).

**H2-a**: Participants presented with a *decoy*_*recipe* (*decoy-comp*) will perceive the system’s quality as higher compared to participants interacting with a system delivering only a *healthy*_*recipe* (*no-comp*) or a *healthy*_*recipe* next to the top matching recipe (*pref-comp*).

### 6.5 Results

#### 6.5.1 Investigating Recommended Recipes

To assess our hypotheses H1-a and H2-a, we looked at the recipes accepted by users during the recommendation phase. Out of 289 users, 47% accepted the *healthy*_*recipe* across all conditions. The average health score of the recipes accepted by the users (M = 7.85, std = 1.42) was significantly lower (t (290) = 6.59, p < .001) than the health score computed based on the recipes users selected to determine their profile (M = 8.52, std = 0.61). This result shows that Cora managed to convince its users to accept recommendations that were healthier than what they would usually cook.

**FIGURE 5:**
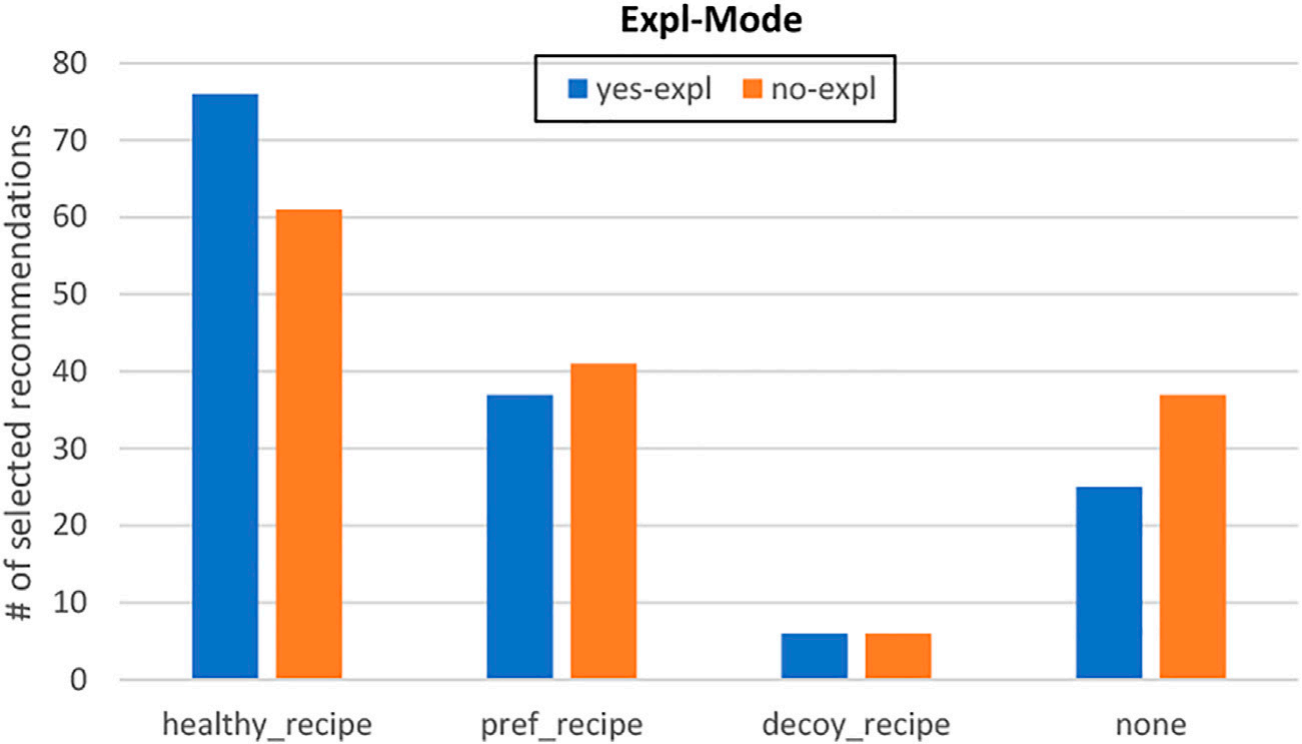
Influence of the explanation mode on accepted recommendations.

**FIGURE 6:**
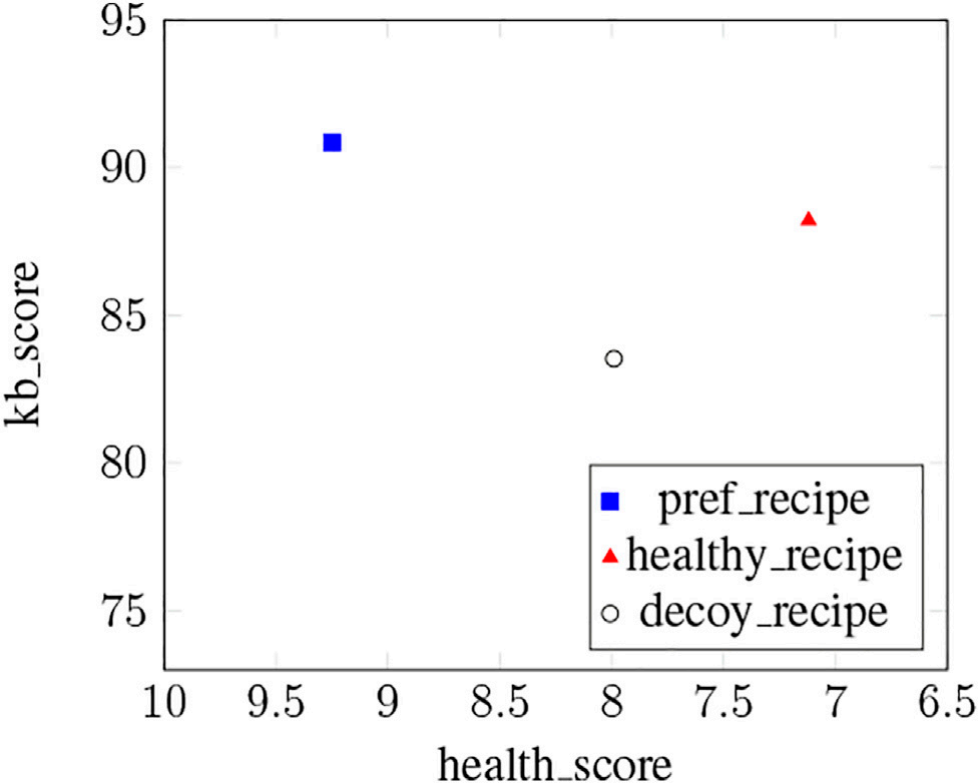
Average *kb*_*score* and *health*_*score* for the three types of recipes recommended by our system.

As shown in Figure 5, explanations positively impacted healthy_recipes’ acceptance rate although the difference was not significant (*χ* ^2^ (3, *N* = 289) = 4.17, *p* = .24). To be sure that Cora generated appropriate decoys, we computed the average *health*_*score* and *kb*_*score* for the recipes recommended in the *decoy-comp* condition. As shown by Figure 6, the *healthy*_*r*_ *ecipes* were healthier and matched more users’ constraints compared to *decoy*_*recipes*, matching the theory (Fasolo et al., 2006).

#### 6.5.2 Quantitative Analysis

To assess our hypotheses H1-b and H-2b, we conducted eight 2x2 factorial ANOVAs (i.e., analysis of variance) with Expl-Mode and Comparison-Mode as between-subject factors.

##### 6.5.2.1 User Beliefs

**FIGURE 7:**
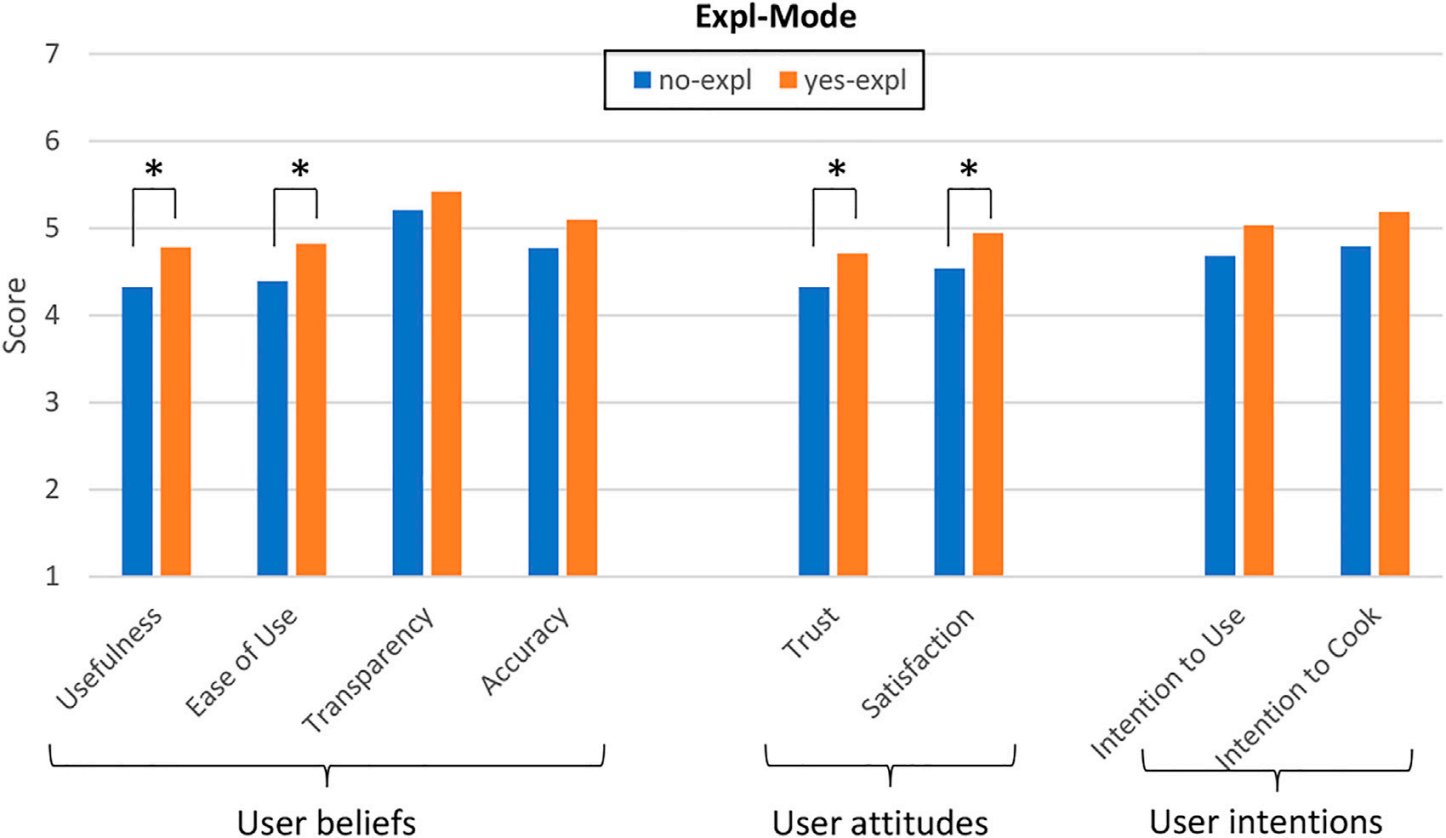
Influence of the explanation mode on user beliefs and user attitudes. The differences between the means are marked according to their level of significance (* for p < 0.05, and ** for p < 0.01).

**FIGURE 8:**
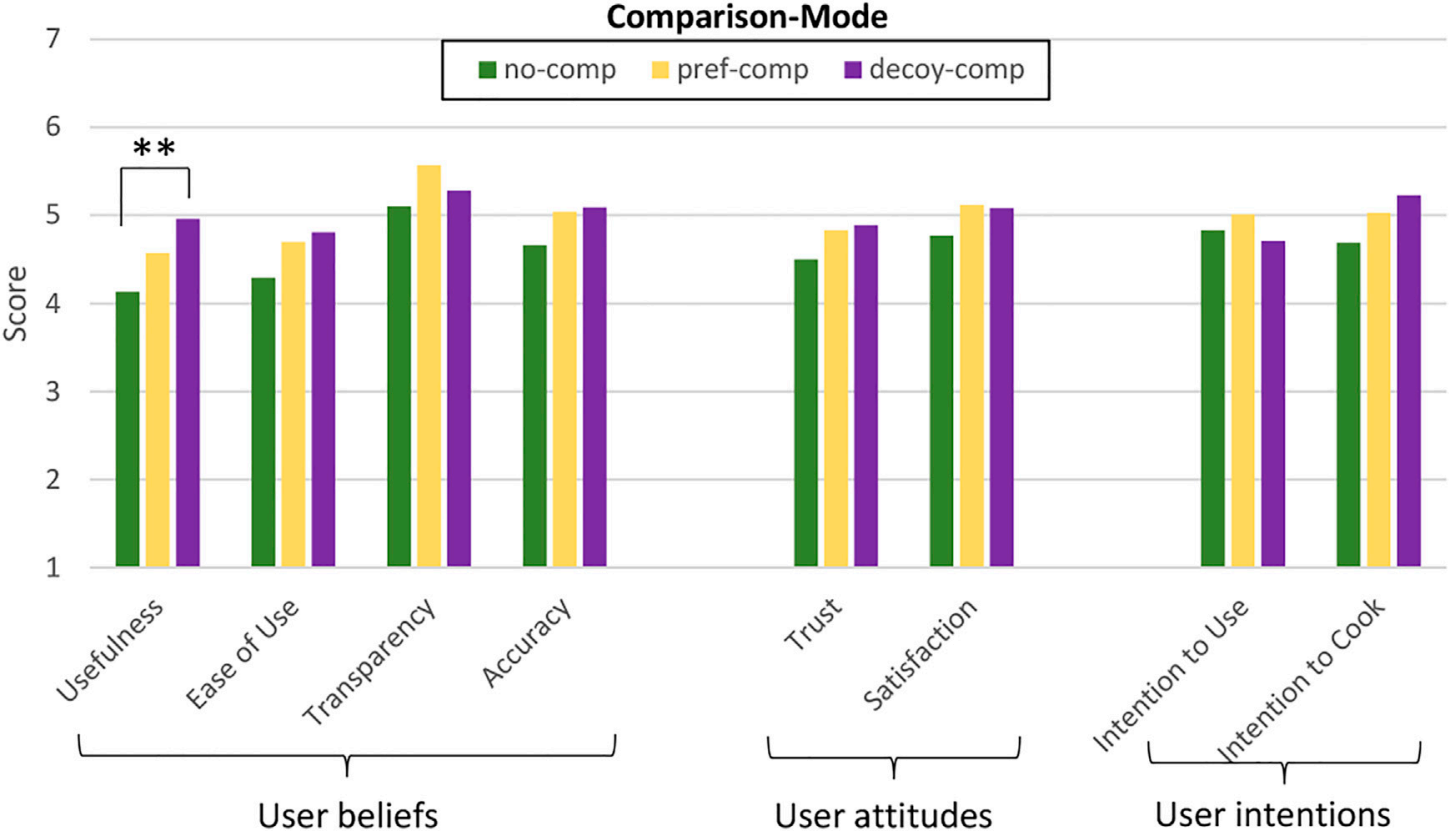
Influence of the comparison mode on user beliefs and user attitudes. The differences between the means are marked according to their level of significance (* for p < .05, and ** for p < .01).

As shown in Figure 7, the version of Cora able to explain its recommendations obtained better scores than the one recommending without explanation. The factorial ANOVAs revealed a significant main effect of Expl-Mode on the perceived usefulness of system (F (1, 283) = 4.564; p < .05) and on the system’s perceived ease of use (F (1, 283) = 4.409; p < .05). Our system Cora was perceived as more useful and easier to use when it justified its recommendations with a specific explanation. There was also a main effect of Comparison-Mode (F (2, 283) = 4.943; p < .01) on the perceived usefulness of the system. The results of the posthoc analysis (after Bonferroni correction) presented in Figure 8 showed that the system was perceived significantly more useful in the *decoy-comp* condition than in the *no-comp* condition (p < 0.01).

As for user beliefs, the version of Cora able to explain its recommendations obtained better scores than the one recommending without explanation (see Figure 7). The factorial ANOVAs revealed a significant main effect of Expl-Mode on users’ trust (F (1, 283) = 4.118; p < .05) and on users’ overall satisfaction with system (F (1, 283) = 4.452; p < .05). Participants were significantly more satisfied with Cora and trusted Cora significantly more when it accompanied its recommendations with an explanation.

##### 6.5.2.3 Behavioral Intentions

**FIGURE 9:**
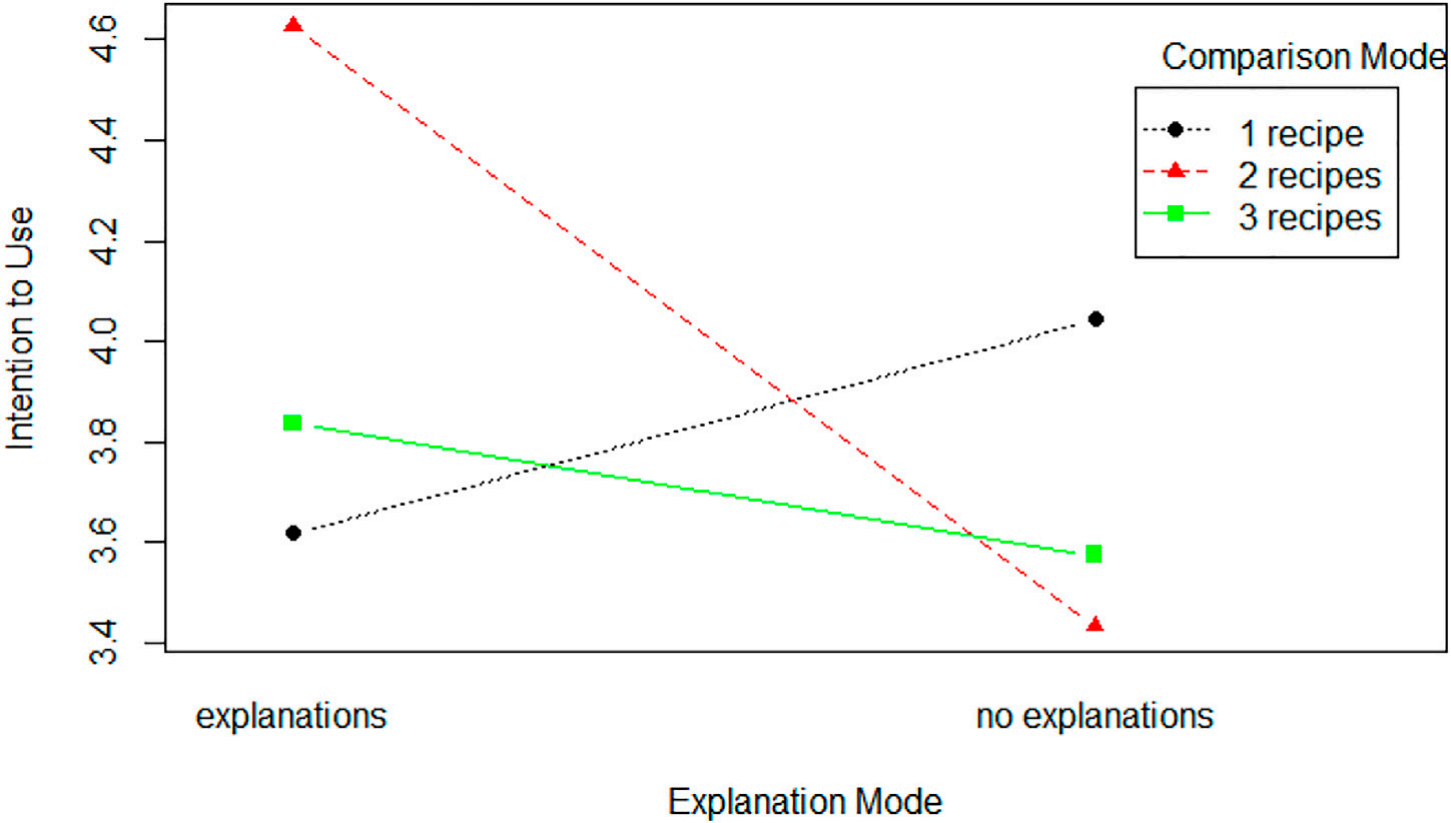
Interaction between explanation mode and comparison mode on users’ intention to use Cora.

The factorial ANOVAs revealed an interaction between Expl-Mode and Comparison-Mode (F (2, 283) = 4.709; p < .01) related to participants’ intention to use the system. Our post-hoc analysis revealed, after Bonferroni correction, that participants in the *pref-comp* condition reported they were more likely to use Cora when it delivered explanations compared to when it did not deliver any explanation (p < 0.05). The interaction is depicted in Figure 9.

#### 6.5.3 Qualitative Analysis

More than half of the participants (51%) left a specific comment at the end of the experiment. The average length of these comments is (m = 23 words; std = 25 words). Overall, 50% of the comments can be classified as positive (e.g., “*I really liked it, very knowledgeable app, makes life easy*”), 30% are considered negative (e.g., “*Not a good search engine. Very behind google*”) and 20% are both (e.g., “*i guess is very practical, and i like the recipe given, but i was looking more for dinner options than dessert*”).

Amongst the positive comments, most of them reveal how easy that was to interact with Cora. Some examples are: *“I enjoyed chatting with Cora. The questions were refreshing and phased in a manner that made it easy to achieve a suitable result.”* (P85), *“It was a really good interaction. Easy, simple and efficient.”* (P285), *“very easy to use and straight forward.”* [P156], or *“Chatting with Cora was very pleasant and easy, both fast and thorough.”* (P269). Many comments state how participants would be happy to use Cora, such as *“it was great I would definitely use Cora for recommendations”* (P117), or “Interested in the finished product, if it ever happens.” (P135). Participants also positively commented on system’s accuracy *“It seems very advanced and receptive of people’s different tastes in food.”* (P129) or explanations *“it was fun, the feedback was relevant and useful”* (P90). Some participants especially appreciated the health-aware aspect of the recommender system: *“Anything helping people to eat more healthy is a good thing.”* (P129), while others expressed their intention to cook the recipe they selected *“I love it! It was great! I saved the recipes and will try them out! Many thanks!”* (P30).

Some of the negative reactions were expected, since they were related to our experimental conditions. For instance, participants in the *no-expl* condition specifically pointed out the lack of explanation, saying that *“Cora was limited. She should have advised on the health benefits more”* (P189). Participants in the *no-comp* condition complained about the lack of alternatives: *“It would have been good if Cora provided a number of recipes in the first instance. A variety of perhaps 5 different ones to choose from.”* (P203), *“Cora gave me only one recipe, based on my preferences. that’s not a lot.”* (P55), or *“maybe Cora should propose several dishes, and a starter/dessert option?”* (P186). Some participants in the *decoy-comp* condition specifically complained about the decoy recipe they were recommended *“I told her I was vegetarian and she gave me a bacon recipe*(*”* (P16) or *“the recipes suggested to me contained one recipe that was not vegan”* (P225). Some participants wanted to have more control over the recommendation, and wanted to add specific constraints to the recommendation process. For example *“Maybe ask what occasion you are cooking eg regular week day meal or a special occasion”* (P57), or *“It was great but I wish there was a question of a kind of meal I would like to cook (dessert, dinner, lunch etc).”* (P60).

Finally, most of the comments related to the interaction design were balanced. Some participants did not enjoy the conversation *“It is just like talking to a robot”* (P66), *“Cora’s responses are bit stiff/feels very AI-ish”* (P237), while some others *“enjoyed chatting with Cora.”* (P85). The modality of the interaction also triggered balanced opinions: *“I would like to type instead of selecting from menu or lists”* (P66) versus *“I thought it was natural and liked choosing items from a dropdown instead of typing and guessing at what she would understand.”* (P226).

## 6.6 Discussion

Explanations had an overall positive effect on users’ perception of the system. Participants found that Cora was significantly easier to use and significantly more useful when it justified its recommendations. Participants were also significantly more satisfied by the recommendations, meaning that our hypothesis H1-b is partially validated. Surprisingly, we did not find any main effect of Expl-Mode on Transparency. That can be explained by the fact that our explanations focused on the healthiness of the recipes while participants might have expected to learn how the recommended recipes matched their preferences and constraints. The presence of a decoy also probably confused participants. Explaining that a recommended item is worse in every aspect than another one defeats the initial purpose of the decoy recipe which plays with a quick and reflexive decision making. Although participants were more likely to select healthy recipes when Cora delivered recommendations with explanations, the results were not significant. Hence, our hypothesis H1-a was not validated. One possible way to solve this problem would be to force participants to choose one recipe instead of allowing them to select none, as it was done in (Fasolo et al., 2006). However, the fact that some participants were not satisfied by any of the recommendations delivered means that our recommendation algorithm should be improved. Forcing people to select the recipe they dislike the least would hide the limitations of our recommendation process.

One issue with our approach is related to the way we build our user profile. Indeed, selecting five recipes out of thirty might not be sufficient to capture people’s eating habits. Another limitation comes from the dataset we collected. Indeed, only 2.3% of the recipes it contains are considered healthy (*health*_*score* < 8), which drastically reduces the number of healthy recipes the system can recommend. A larger dataset containing more healthy options would make the system more accurate and appropriate for the goal of improving eating habits. Another way to refine Cora’s recommendations would be to ask more questions during the interview phase, including the type of meal they would like to eat (e.g., dinner, dessert, meal, etc … ) or the type of cuisine they would like to eat (e.g., Italian, Indian, Korean, etc … ).

The effects of the comparison mode on users’ perception were mixed. Although recommending alternatives to the participants had a positive impact overall, our results combined with users’ feedback suggest that the decoy option should be selected carefully. The lack of significant differences for most of the items except one shows that adding an option that does not match users’ constraints might have a negative impact on the perceived accuracy of the system, as well as on users’ trust and satisfaction.

Overall, combining comparisons with a decoy and explanations worked well as users in the *decoy-comp* and *yes-expl* condition gave higher scores to the system for user beliefs and user attitudes. However, the graph in Figure 9 gives us interesting insights on how the presence of explanations interacts with the number of recipes compared by Cora regarding participants intention to use. Indeed, participants reported that they would be significantly more likely to use Cora in the future if it delivered no more than two alternatives and explained the difference between them. Although adding a decoy might be interesting for a single interaction, limiting the number of recommendations delivered at the same time might be better in the long run.

Another interesting result is linked to the design of the interaction. As explained in Section 6.5.3, our design choices were not generally accepted by participants. This is consistent with the feedback collected during our first experience. Some users actively wanted to engage in a discussion with the recommender system, while others simply wanted to get a recommendation in the most efficient way. Some users would have preferred to type instead on using buttons, while others were happy with this modality.

## 7 Conclusion

In this paper, we presented Cora, a conversational recommender system able to recommend recipes matching users eating habits and needs. Cora was able to engage its users in a rapport-building dialogue or a task-oriented one, and was able to interact with them using free-text or buttons and drop-down menus. Cora was also able to propose different alternatives and to justify its recommendation by explaining the trade-off between them.

We used Cora in two different experiments whose goal was to explore the design of persuasive conversational systems for promoting healthy eating behaviors. We conducted a first user study to evaluate the influence of Cora’s conversational skills and users’ interaction mode on users’ perception and intention to cook. Our results show that endowing conversational recommendation systems with rapport-building conversational strategies significantly improves users’ perception of the interaction and of the system itself. We also found that rapport-building strategies were a way to mitigate and lower the impact of the system’s misunderstandings on users perception and intention to cook.

We built on the results we obtained during our first experiment to improve Cora’s recommendation process and the design of the interaction. We then conducted another user study to evaluate the influence of Cora’s explanations and recommendation comparisons on users’ perception of the system. In the second evaluation, we evaluated the influence of Cora’s explanations and recommendation comparisons on users’ perception of the system. Our results show that explanations positively influence users’ perception of a recommender system. However, comparing healthy recipes with a decoy was a double-edged sword. Although such comparison was perceived as significantly more useful compared to one single healthy recommendation, explaining the difference between the decoy and the healthy recipe actually made people less likely to use the system. Moreover, our results showed that Cora was able to convince its users to accept recommendations that were healthier than what they would usually cook.

One potential extension of this work is to develop a mobile application to investigate whether the nudges we implemented could still be effective in the long run. A longitudinal study would allow our system to ask follow-up questions whenever users reject a recommendation to understand why they rejected it. That would help the system to refine its users’ profiles and to deliver more accurate recommendations later on (Chen and Pu, 2009).

## Data Availability

The raw data supporting the conclusion of this article will be made available by the authors, without undue reservation.

## Footnotes

1 The health score can be calculated following the United Kingdom Food Standard Agency guidelines https://assets.publishing.service.gov.uk/government/uploads/system/uploads/attachment_data/file/566 251/FoP_Nutrition_labelling_UK_guidance.pdf

2
https://spoonacular.com/food-api

## Supplementary Material

Supplementary Figure S1 Influence of Cora Mode on the perceived quality of the interaction. The differences between the means are marked according to their level of significance (* for p < 0.05, ** for p < 0.01, and *** for p < 0.001).

Supplementary Figure S2 Influence of User Mode on the perceived quality of the interaction.

Supplementary Figure S3 Influence of Cora Mode on the perceived quality of the system. The differences between the means are marked according to their level of significance (* for p < .05, ** for p < 0.01, and *** for p < 0.001).

Supplementary Figure S4 Influence of User Mode on the perceived quality of the system.

Supplementary Figure S5 Influence of Cora Mode on users’ intention to cook.

Supplementary Figure S6 Influence of User Mode on users’ intention to cook.
