# Supplementary figures and images for: Designing Persuasive Food Conversational Recommender Systems With Nudging and Socially-Aware Conversational Strategies
Source: Front Robot AI. 2022 Jan 19;8:733835. doi: 10.3389/frobt.2021.733835 (PMC8807554; doi:10.3389/frobt.2021.733835)

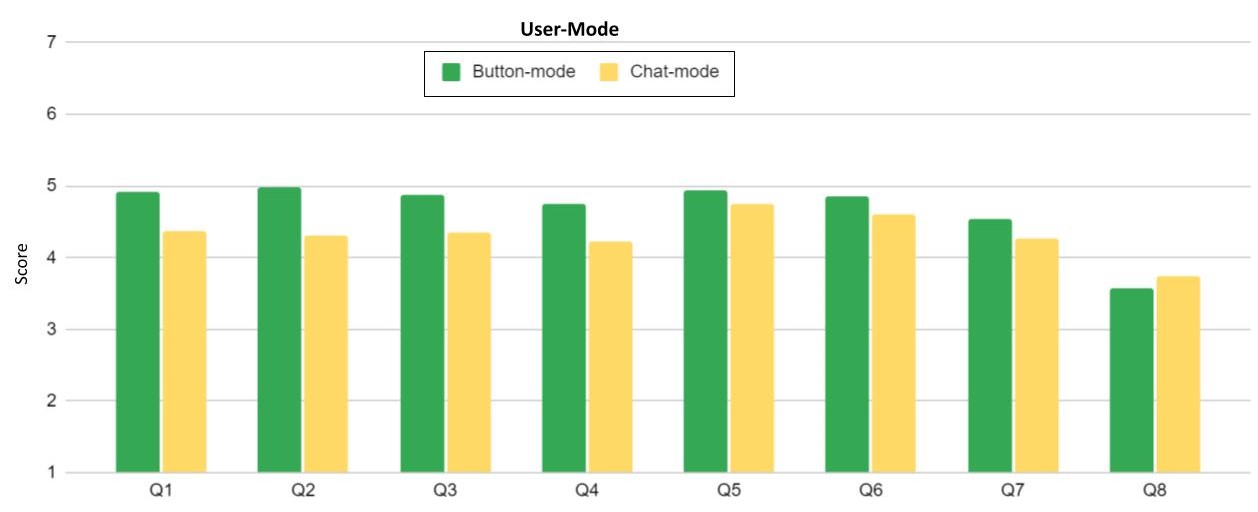

Supplement: Supplementary file 1 [file Image5.jpg]

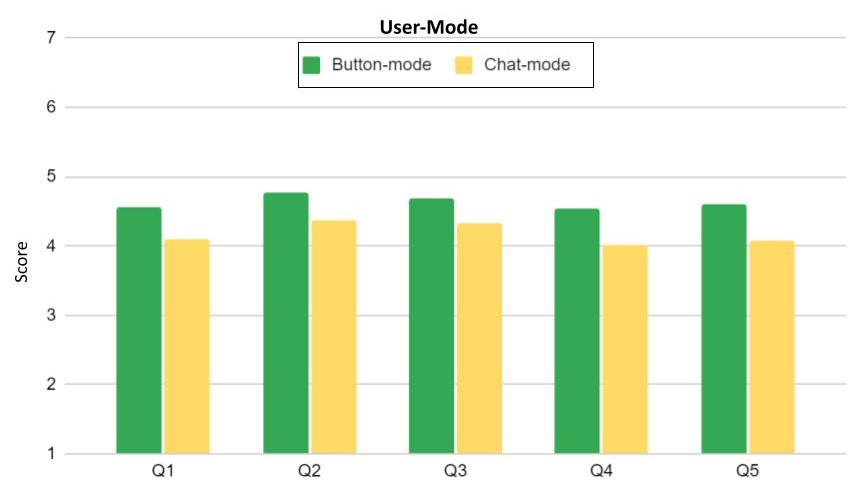

Supplement: Supplementary file 2 [file Image6.jpg]

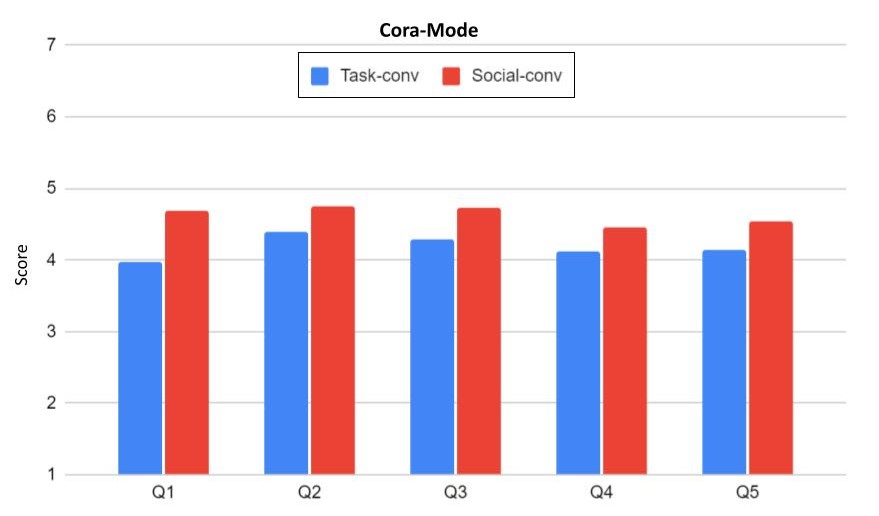

Supplement: Supplementary file 3 [file Image3.jpg]

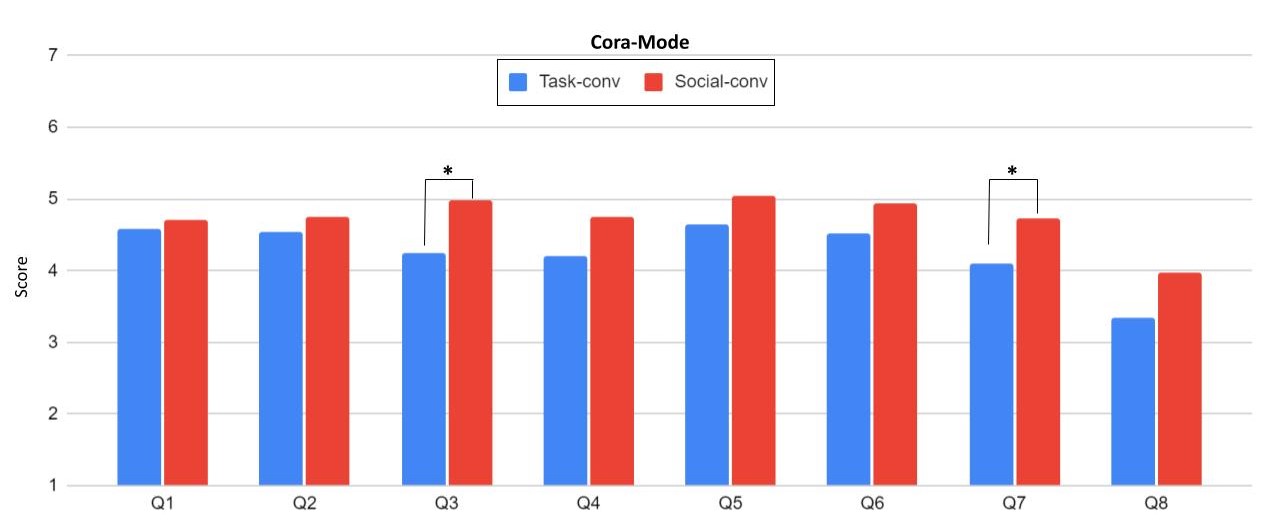

Supplement: Supplementary file 4 [file Image2.jpg]

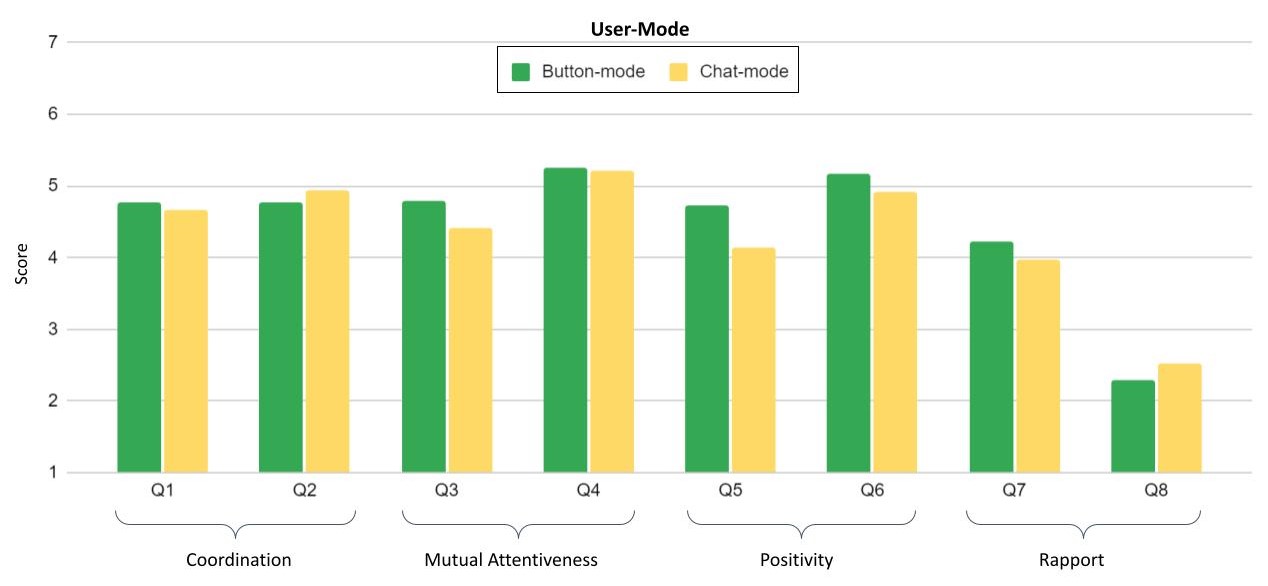

Supplement: Supplementary file 5 [file Image4.jpg]

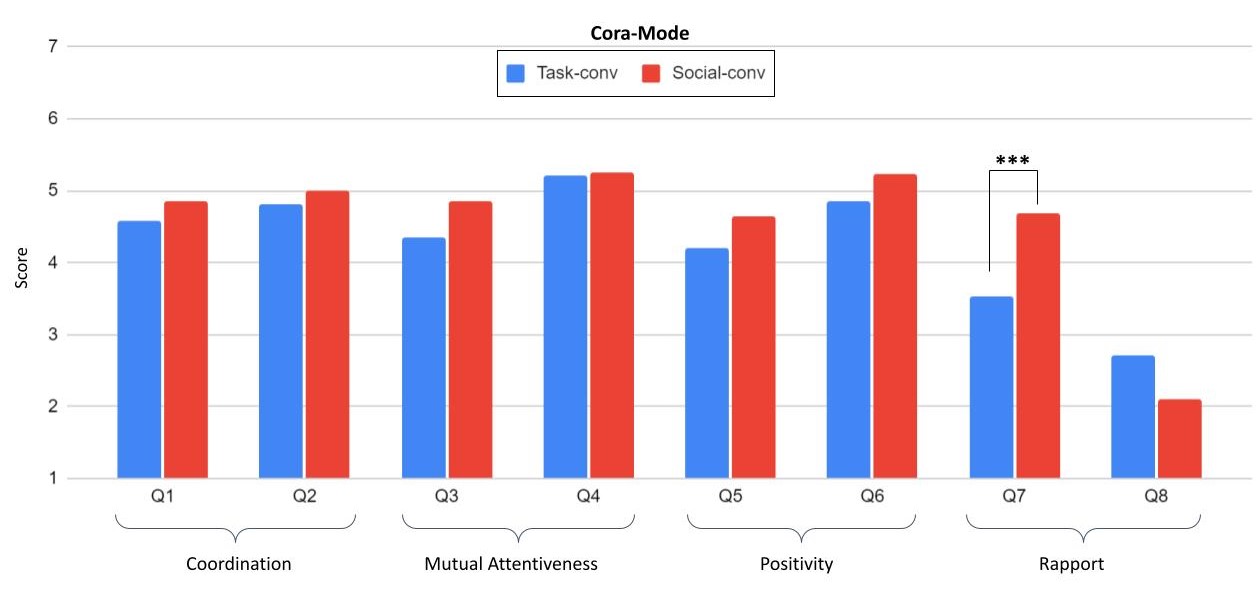

Supplement: Supplementary file 6 [file Image1.jpg]
